# Review of Microfluidic Devices and Imaging Techniques for Fluid Flow Study in Porous Geomaterials

**DOI:** 10.3390/s20144030

**Published:** 2020-07-20

**Authors:** Amir Jahanbakhsh, Krystian L. Wlodarczyk, Duncan P. Hand, Robert R. J. Maier, M. Mercedes Maroto-Valer

**Affiliations:** 1 Research Centre for Carbon Solutions (RCCS), School of Engineering and Physical Sciences, Heriot-Watt University, Edinburgh EH14 4AS, UK; K.L.Wlodarczyk@hw.ac.uk (K.L.W.); M.Maroto-Valer@hw.ac.uk (M.M.M.-V.); 2 Institute of Photonics and Quantum Sciences, School of Engineering and Physical Sciences, Heriot-Watt University, Edinburgh EH14 4AS, UK; D.P.Hand@hw.ac.uk (D.P.H.); R.R.J.Maier@hw.ac.uk (R.R.J.M.)

**Keywords:** microfluidic devices, micromodels, imaging techniques, porous media, geomaterials, pore-scale, transport phenomena, geoscience, geo-energy engineering

## Abstract

Understanding transport phenomena and governing mechanisms of different physical and chemical processes in porous media has been a critical research area for decades. Correlating fluid flow behaviour at the micro-scale with macro-scale parameters, such as relative permeability and capillary pressure, is key to understanding the processes governing subsurface systems, and this in turn allows us to improve the accuracy of modelling and simulations of transport phenomena at a large scale. Over the last two decades, there have been significant developments in our understanding of pore-scale processes and modelling of complex underground systems. Microfluidic devices (micromodels) and imaging techniques, as facilitators to link experimental observations to simulation, have greatly contributed to these achievements. Although several reviews exist covering separately advances in one of these two areas, we present here a detailed review integrating recent advances and applications in both micromodels and imaging techniques. This includes a comprehensive analysis of critical aspects of fabrication techniques of micromodels, and the most recent advances such as embedding fibre optic sensors in micromodels for research applications. To complete the analysis of visualization techniques, we have thoroughly reviewed the most applicable imaging techniques in the area of geoscience and geo-energy. Moreover, the integration of microfluidic devices and imaging techniques was highlighted as appropriate. In this review, we focus particularly on four prominent yet very wide application areas, namely “fluid flow in porous media”, “flow in heterogeneous rocks and fractures”, “reactive transport, solute and colloid transport”, and finally “porous media characterization”. In summary, this review provides an in-depth analysis of micromodels and imaging techniques that can help to guide future research in the in-situ visualization of fluid flow in porous media.

## 1. Introduction

Porous media contain a complex network of interconnected pores that allow fluids to flow through a porous medium. These characteristics are encapsulated within two macroscopic parameters, namely effective porosity (ϕ) and permeability (k). Effective porosity (ϕ) is the ratio of connected void space to the total volume of the rock, whilst permeability is the capacity to transmit fluids. The classification and evaluation of underground geological formations are based on these parameters. A reservoir with moderate porosity and permeability has ϕ and k values of 15–25% and 50–500 millidarcy (mD), respectively [1]. Moreover, ϕ and k are affected by the size, shape, arrangement, connectivity and tortuosity of pores [1]. Upscaling the governing mechanisms from small to large scale is very challenging and requires an understanding of complex physical and chemical processes that occur over a range of scales, from pore-scale (microscopic, nm to µm) to lab-scale (macroscopic, mm to cm) and field-scale (reservoir, km) [2].

Traditionally, to gain information about flow and investigate its governing physical and chemical mechanisms, indirect measurements have been used. The common practice is to take a core sample (scale of centimetres) from a geomaterial (namely any material with geological origin, e.g., rocks) and perform a series of flow experiments under reservoir conditions using either original or analogous fluids. By measuring the inflow and outflow rates and the pressure drop across the tested geomaterial at the lab scale, dynamic flow parameters, such as relative permeability (*kr*), can be determined. *kr* is a dimensionless measure of the effective permeability of one fluid phase in presence of other fluid phases. Novel imaging and visualization techniques enable direct measurements and detailed analysis of events even at the pore-scale to provide information about the fluid front evolution and saturation distribution [3,4,5,6]. Moreover, two- and three-dimensional (2D and 3D) imaging techniques enable qualitative and quantitative analysis, as well as the validation of mathematical models. Flow visualization using microfluidic devices has provided significant breakthroughs in monitoring and capturing flow behaviour and mechanisms [7,8,9,10,11] that can be used for qualitative interpretation and to some extent for quantitative analysis. 

It is essential to understand the current and potential applications of microfluidic devices and imaging techniques for research in geoscience, environmental and geo-energy engineering. Several reviews exist in the areas of microfluidic devices [7,8,9,10] and visualization techniques [3,4,12,13,14]. This review, however, uniquely evaluates both microfluidic devices and imaging techniques focusing on pore-scale applications and potential links to continuum scale flow parameters. Fabrication methods of micromodels using different materials, such as photoresists, transparent polymers, glass, silicon, and geomaterials are thoroughly reviewed in Section 2. Similarly, most commonly used imaging techniques, such as high-resolution cameras, optical microscopy, 3D and 4D X-ray (micro) computed tomography (CT/µCT), neutron tomography, positron emission tomography (PET), nuclear magnetic resonance (NMR), magnetic resonance imaging (MRI), single- and dual-energy gamma radiation, focused ion beams (FIB), scanning electron microscopy (SEM), and transmission electron tomography (TEM) are critically discussed in Section 3. The applications of these visualization techniques for “fluid flow in porous media”, “flow in heterogeneous rocks and fractures”, “reactive, solute, and colloid transport” and “porous media characterization and rock/soil deformation” in geoscience, petroleum engineering, geo-energy engineering, and hydrogeology are also reviewed. This manuscript provides extensive and useful guidelines to readers for selecting the proper research methodology and techniques for investigating subsurface processes happening in porous media, including the selection of the right material and manufacturing method for micromodel fabrication as well as employing the most compatible imaging technique for either micromodel testing or core sample experimentation.

## 2. Microfluidic Devices (Physical Micromodels of Porous Geomaterials)

This section provides a critical overview of different fabrication methods of microfluidic devices used for the investigation of different physical and chemical processes occurring in subsurface systems. This includes a description of various manufacturing techniques using different materials, together with relevant applications of these devices to study processes such as carbon dioxide (CO_2_) storage, gas trapping, enhanced oil recovery, and dissolution of chemical substances, minerals and contaminants in porous media and hydrogeology.

In general, microfluidic devices can be defined as 2D or 3D enclosed microstructures to which access is provided by at least one pair of holes serving as inlet and outlet ports. The complexity of the microstructures can be diverse. Very simple microstructures may contain only a single microchannel, whereas very complex ones may contain a large number of microchannels of various dimensions, complex levels of interconnections, as well as integrated micro-pumps, micro-valves, micro-mixers, micro-heaters, etc. Such complex microfluidic devices are often called “lab-on-a-chip” systems. 

**Figure 1:**
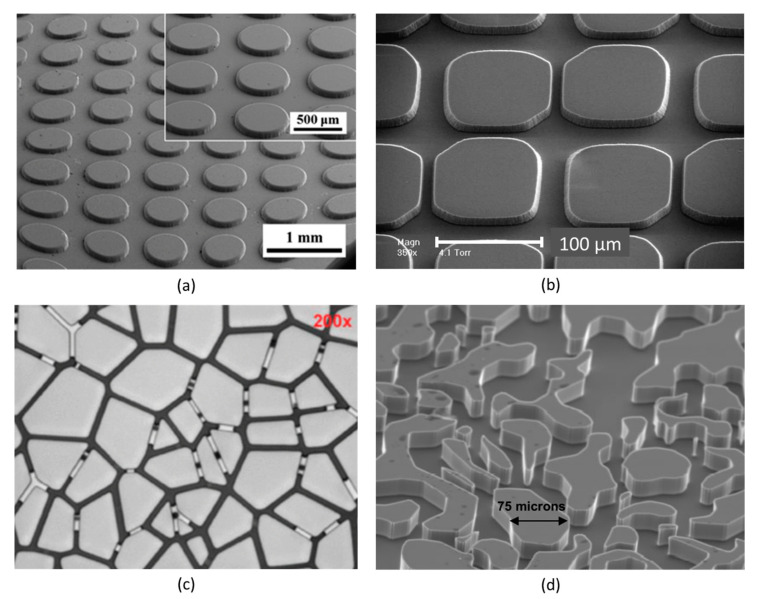
Examples of different pore network patterns: (**a**) regular (from Hsu et al. [15]), (**b**) partially-regular (from Auset & Keller [16]), (**c**) quasi-irregular (Xu et al. [17]), and (**d**) irregular (from Rangel-German & Kovscek [18]). (Reprinted with permission from [15,16,17,18]).

Microfluidic devices have found a wide range of applications in many industrial and research areas, primarily in chemistry, biology, medicine and pharmacology, enabling the direct observation and investigation of various physical, chemical, and biological processes occurring at small (even submicron) scales. These devices have also found use in geoscience, hydrogeology, petroleum and geo-energy engineering research to conduct experimental investigations of various processes occurring in porous media. These physical micromodels contain an artificial structure of interconnected pores whose shapes are designed in such a way to represent simplified geometries of geomaterials, such as rocks and core plugs. The internal structure of the micromodels, as shown in Figure 1, can contain a regular, partially regular, quasi-irregular (fractal), or irregular pore network patterns [8]. In regular patterns, all pores and throats have almost the same geometry and dimensions throughout the whole network. However, pores and throats dimensions are variable in partially regular patterns although they form a regular lattice. The quasi-irregular patterns follow the rules of percolation theory and for flow to happen the minimum porosity of 50% is required for a correlated network. The geometry of irregular networks has no spatial correlation and pores are randomly placed. However, the pore sizes follow a statistical distribution. The pore network patterns extracted from real rock samples can be categorized as irregular patterns [8,15,16,17,18].

Generally, porous media micromodels have been proven to be suitable tools for conducting small-scale experiments that provide the opportunity to discover yet unrecognized processes and enhance the understanding of existing theories and assumptions [19].

The following section provides an in-depth analysis of the different types of microfluidic devices manufactured from a wide range of materials to replicate internal structures of various geomaterials. This includes the review of various fabrication techniques (i.e., methods used for the generation and enclosure of pore network patterns), as well as a critical analysis of the advantages and limitations for each method. Table 1 provides a concise summary of the models manufactured to replicate various geomaterials that are analysed in this review.

### 2.1. Photoresist-Based Models 

**Figure 2:**
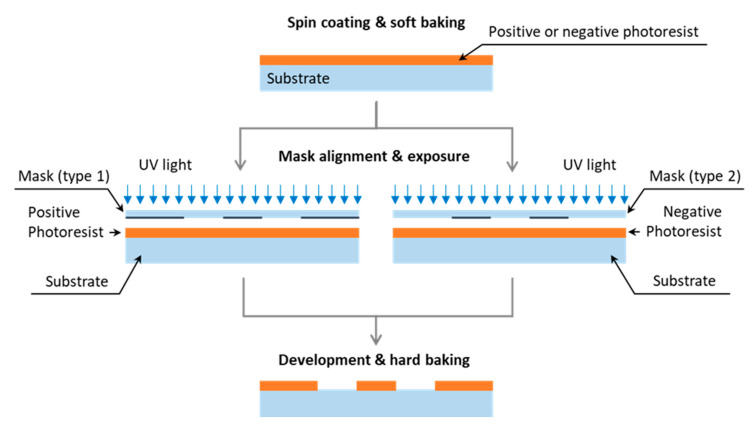
Photolithography process.

Porous media models made of a photoresist are manufactured by photolithography, i.e., the same technology that was primarily developed for the manufacture of integrated circuits [20,21]. The principles of photolithography are presented in Figure 2 and briefly described below.

The first step of photolithography involves applying a photoresist onto a flat and clean substrate (typically glass or silicon, less often polymers such as poly-methyl-methacrylate (PMMA)) by spin coating. The photoresist is then pre-hardened in the so-called ‘soft baking’ process. The final thickness of the photoresist layer, which defines the final depth of pores and channels in the model, depends on the type of the photoresist used as well as the duration and speed of spinning. Both positive and negative photoresists, as shown in Figure 2, can be used for the generation of pore network patterns. The patterns are digitally generated using appropriate software and then transferred onto a mask, which can be made of either a transparency or a metal-coated glass substrate. Patterns on transparencies are generated by printing, whereas patterns on metal-coated glass substrates are generated by removing selectively the metal layer with the use of a laser or an electron beam writer. The masks made of metal-coated glass substrates enable the generation of micro-channels and pores in the range of a few μm [22]. These masks, however, are significantly more expensive (20–100 times) than those produced on transparencies. The next steps of photolithography involve the mask alignment and the photoresist exposure to UV light. The exposure method as well as the distance between the mask and the photoresist have a significant impact on the minimum feature sizes that can be generated. The wavelength of the light source (typically λ = 350–430 nm) also affects the minimum size of features generated on a photoresist. The photoresist development and the so-called ‘hard baking’ are the last two steps of photolithography. When a positive photoresist is used, the UV exposed areas interact with a developing agent and they are washed away, as shown in Figure 2. What is left forms the designed porous pattern. For a negative photoresist, the situation is the same but valid for the unexposed area. The ‘hard baking’ process is necessary to harden the photoresist and enhance its adhesion to the substrate.

Following the photolithography process, the pore network structure must be closed from the top. The cover plate is typically made of a transparent material (either glass or PMMA). Prior to the bonding process, the plate is coated with a photoresist (from one side) which is then ‘soft baked’. The soft photoresist acts as a glue that bonds the cover plate with the plate containing a micro-structure made of the ‘hard’ photoresist. The bonding is accomplished by pressing the two plates together. The following UV exposure and ‘hard baking’ are performed to increase the strength of the bonds.

The fabrication process of photoresist-based porous network models is relatively straightforward and inexpensive. Unfortunately, there are a couple of issues related to the fabrication and usage of these models, as highlighted in Table 1. Firstly, the bonding strength in these models is not very high, particularly when the flow network pattern contains areas which are too small to act as bonds between the two plates containing a photoresist. The second problem emerges from the photosensitive nature of photoresists. Although a photoresist is hardened during the photolithography process, this material may undergo gradual degradation by N_2_ bubbles that are generated within the photoresist while exposed to light, in particular when the wavelength is near the violet or UV spectrum [23]. This undesirable effect often leads to the destruction of the pore network pattern within the model [8].

### 2.2. Polymer-Based Models

Transparent polymers, such as poly-di-methyl-siloxane (PDMS), poly-methyl-methacrylate (PMMA), and cyclic olefin copolymer (COC), are significantly cheaper than silicon and glass. Pore network structures can be generated on these materials simply by moulding or embossing, whereas the sealing of these structures can be performed by using adhesives. Unfortunately, there are a few disadvantages in using polymers as substrates for microfluidic devices. Firstly, some processes used in the manufacture of polymer microfluidics can lead to significant changes in the surface chemistry of these materials, affecting their wetting properties. Therefore, additional processes are often required to recover and stabilise their natural wettability state. Secondly, some polymers are incompatible with organic solvents and low molecular weight organic solutes, and hence the number of applications for this type of microfluidic devices is limited in comparison to the glass-based and silicon-based models (see Section 2.3.2 and Section 2.4 for more details). In addition, polymers are generally incompatible with high temperatures, and often they cannot cope with differential pressures (ΔP) higher than 400 kPa (4 bars).

#### 2.2.1. PDMS Models

PDMS is an optically transparent, silicon-based, elastomeric polymer that has been widely used for the construction of microfluidic devices. The PDMS-based porous media models are fabricated by soft lithography [16,22,24,25,26,27]. The first step of soft lithography involves the development of a master that later is used for the replication of pore network structures on PDMS. The master can be generated, for instance, by photolithography, photolithography and chemical etching, direct laser writing, or conventional (mechanical) machining. Photolithography (described in Section 2.1) is the most common technique used for the generation of masters because it provides high spatial resolution and surface finish. In this technique, a photoresist (usually SU-8) is deposited onto a silicon wafer and then is exposed to UV light through a projection mask comprising the desired flow network pattern and the inlet and outlet areas. High-resolution transparencies are typically used as masks for rapid prototyping of patterns on the SU-8 photoresist. The developed master is used for the replication of a pore network structure on PDMS. The replication is typically performed by moulding. In this process, PDMS of low viscosity is mixed with a curing agent, poured onto the master surface, and then cured at an elevated temperature (approximately 333 °K or 60 °C) for a specific period of time (typically 1–2 h). Following the curing process, the PDMS replica is peeled off the master. The access holes (inlets and outlets) are mechanically generated, either by drilling or punching the cured PDMS with a needle. Channels generated in PDMS by moulding can have a width of only a few micrometres [24], if the height to width aspect ratio is close to unity, whereas surface roughness of such channels depends on the surface quality of the mould used for replication.

Transparent plates made of PDMS or glass are used for sealing pore network structures from the top. The sealing process can be either reversible or irreversible. A reversible seal that can be broken numerous times without damaging the PDMS replica is obtained by pressing the cover plate to the PDMS. This type of sealing, which holds two materials together as a result of the van der Walls forces, is fast and watertight but it cannot withstand pressures > 34.5 kPa (0.35 bar) in the capillaries [24,26]. An irreversible seal is usually provided by exposing the PDMS and the cover plate to an oxygen plasma. However, other bonding methods, such as corona discharge, partial PDMS curing, cross-linker variation and uncured PDMS adhesive, can also be used if the cover plate is made of PDMS [28]. The oxygen plasma is believed to generate silanol groups (Si-OH) on the surface of PDMS by oxidizing the methyl groups [24,25,26], which leads to the formation of bonds between the PDMS and the cover plate. The irreversible seal can withstand pressures up to 345 kPa (3.5 bar). The attempt of breaking the seal results in a failure of PDMS. The PDMS replica can be irreversibly sealed to many materials, such as PDMS, glass (including fused silica), silicon, quartz, silicon nitride, polyethylene, polystyrene, and glassy carbon. The irreversible seal, however, cannot be obtained with polyimide, PMMA, and polycarbonate [24,25,26]. 

PDMS is hydrophobic in its natural state with a water contact angle of around 110° [29]. This material, however, can change its wetting properties during the bonding process. For instance, the oxygen plasma technique causes PDMS to become hydrophilic (contact angle for water ≈ 10°), whilst an activation of the PDMS surface by corona discharge makes the polymer more hydrophobic than in its natural state. Fortunately, there are methods for recovering and stabilising the natural PDMS hydrophobicity. For instance, Karadimitriou et al. [27] demonstrated that the hydrophobicity of channels in a PDMS model can be restored by injecting a solution of trichloro-perfluoro-octyl-silane in 96% pure ethanol. Moreover, Schneider et al. [30,31] developed a method to fabricate PDMS micromodels with flow patterns of well-controlled wettability. This allowed them to selectively alter surface wettability of pores and channels and reproduce the wetting heterogeneity that is observed in many hydrocarbon reservoirs. 

There are several advantages of using soft lithography for the generation of microfluidic devices from PDMS. This includes low cost and fast fabrication time (<1 day to final device), reusability of masters, simple sealing procedure of PDMS with a large number of different substrates, as well as the ability to manufacture complex 3D microfluidic devices [25]. High gas permeability of PDMS makes this material also suitable for a variety of biological and cellular applications. As demonstrated by Zhao et al. [32], PDMS can also be used as a cast form to replicate pore network patterns on photocurable polymers, such as Norland Optical Adhesive 81. On the other hand, PDMS is unsuitable for carrying out in-channel oxygen-sensitive polymerization reactions, and often it is not compatible with organic solvents, which limits its use to aqueous solutions [27]. Moreover, the low stiffness of PDMS leads to elastic deformations of micro-channels within the microfluidic devices even under very low flow rates and injection pressures as low as 13.8 kPa (0.14 bar) [17,27,33,34].

Application of PDMS micromodels has been very popular to study the behaviour of single-phase and two-phase fluids in porous media during imbibition and drainage processes [17,27,35] and to investigate various factors governing the transport of colloids in the subsurface systems [16].

#### 2.2.2. PMMA Models

PMMA is an acrylic thermoplastic material which is stiffer and harder than PDMS. In some cases, PMMA is used as a substitute of glass due to its high transparency. Pore network structures on PMMA can be generated by either direct laser writing [22,36,37,38,39,40,41,42,43,44] or using a LIGA process (a German acronym for “Lithographie, Galvanoformung, und Abformung”) [22,44,45,46,47,48]. The first method is preferable for rapid prototyping and manufacture of microfluidic devices in low quantities, whilst the second is suitable for production of identical microfluidic devices in large quantities. 

Direct writing of microfluidic channels on PMMA is typically performed using a KrF excimer laser [22,36,37,38,44]. These lasers are capable of producing nanosecond pulses of wavelength 248 nm, which are well absorbed by the PMMA material, enabling generation of arbitrary structures with high vertical accuracy (<50 nm) and lateral (sub-μm) resolution. Channels generated by a KrF laser can have rectangular cross-sections and vertical sidewalls [44]. The surface roughness (Ra) of these channels is typically in the range of 1–3 μm [36,37]. The very low beam quality of excimer lasers means that they are only suited to a mask-imaging approach which is inflexible and unsuited to low volume manufacture. The other drawbacks of excimer lasers are their high capital and running costs. These lasers require the use of a mixture of expensive and hazardous gases to provide emission of the laser beam and specialised UV optics for the laser beam delivery.

CO_2_ lasers can also be used for the generation of microfluidic channels on PMMA [39,40,41,42,43], but these lasers do not provide as high machining resolution as excimer lasers. The CO_2_ lasers (λ ≈ 10.6 μm) enable the generation of channels with very low surface roughness (Ra < 10 nm), but this advantage is achieved at the cost of the reduced machining resolution [41]. Micro-channels generated by a CO_2_ laser beam are relatively wide (typically > 150 μm), and they possess a rounded bottom and sloped sidewalls [39,40,41]. As shown by Cheng et al. [40], it is possible to create micro-channels with aspect ratios > 7. 

Another laser that is used for the generation of microfluidic channels on PMMA is a frequency doubled Ti: sapphire femtosecond laser (λ = 400 nm) [42]. This laser is capable of producing channels on PMMA without burr formation, enabling straightforward, high quality sealing of the microfluidic channels by using another PMMA plate. Channels 40–100 μm wide and 50–300 μm deep usually have moderate surface roughness (Ra = 0.55–0.75 μm). Like the CO_2_ laser-generated channels, they also possess sloped sidewalls and a rounded bottom.

**Figure 3:**
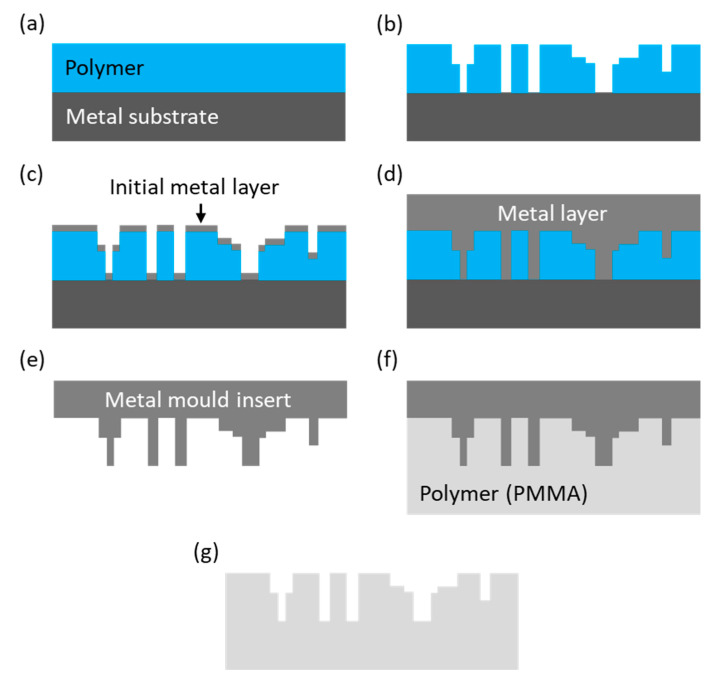
Scheme of the process sequence in LIGA: (**a**) spin coating and baking, (**b**) microlithography or direct laser writing, (**c**,**d**) electroplating, (**e**) machining, separation and dissolution of the polymer, (**f**) injection moulding or embossing, and (**g**) demoulding.

The LIGA technique is used when a high reproducibility of microfluidic patterns is required in a large quantity. This fabrication method involves a sequence of three processes: deep (X-ray or UV) lithography, electroplating, and moulding [22,44,45,46,47,48]. Figure 3 shows the sequence of processes employed in LIGA.

In the first step, a metal substrate is covered with liquid polymer (either photoresist or PMMA monomer), which later is thermally polymerised in a baking process. Following this step, the designed pore network pattern is transferred onto the hardened polymer either by X-ray or UV lithography [44,45,46] or by direct laser writing using an excimer laser [47,48]. X-ray lithography is suitable for PMMA because this material has a strong absorption near 1 nm. The X-rays enable the generation of narrow and smooth channels with a high aspect ratio and almost vertical sidewalls. On the other hand, X-ray lithography requires the use of customised masks (made of quartz and chrome, or Kapton™ and gold) which makes this process expensive and time consuming, in particular for the manufacture of micromodels in a low quantity [44]. Following the lithography (or direct laser writing) process, the etched polymer is coated with a thin layer of metal (e.g., nickel) to initiate electroplating, as shown in Figure 3c. The plating process is carried out to the point where the metal layer covers the whole micro-structure and is thick enough to be fitted to a moulding or embossing tool. The metal mould insert is then separated from the polymer by shock-freezing using liquid nitrogen. Following this process, the insert is cleaned from residual polymer by dissolving in organic solvents. Such a prepared metal mould insert is ready for the replication of pore network patterns on PMMA by either injection moulding or hot embossing. The micro-structures generated by the LIGA process are typically closed from the top with a second PMMA plate by either thermal bonding or adhesives [39,40,41,48,49]. 

#### 2.2.3. COC Models

Recently, it has been demonstrated that porous media micromodels can also be manufactured from a polymer called cyclic olefin copolymer (COC) [15]. This rigid thermoplastic material is characterised by high optical transmission in the UV and visible spectrum range, low water absorption, good resistance to many acids, and exceptionally good resistance to a host of solvents including organics (e.g., acetonitrile) [49]. 

The COC-based porous media model described by Hsu et al. [15] contained a matrix of cylindrical pores, which was divided into two by an approximately 2.6 mm wide channel, with two secondary micro-channel outlets on both sides. This complex microstructure was fabricated by a hot embossing process. A UV/Ozone treatment was used for sealing the pore network structure as well as for modifying the surface wettability of pores and channels. The pores in the micromodel were 500 μm in diameter and 100 μm deep, and thus they were larger than pores in many real geomaterials. The 2.6 mm wide channel across the pore network area represented a fracture inside a natural rock.

#### 2.2.4. Resin-Based Three-Dimensional (3D) Printing 

Additive manufacturing (AM), also called 3D printing, is a modern technology that enables rapid prototyping and manufacturing of 3D objects from a digital file. In AM processes, successive layers of materials are laid down on top of each other until a 3D object is formed [50]. In comparison to traditional manufacturing techniques, 3D printing has several advantages for fabricating micromodels, including relative fast prototyping, increased part complexity, cost effective, and waste reduction. However, mass production is still the main disadvantage of 3D printing [51]. Moreover, 3D printing has a wide range of applications in research, education, engineering, architecture, construction, and many others. In geoscience research, 3D printing technology has created new opportunities. Producing rock proxies, i.e., replicating a rock sample by preserving its internal structure e.g., pore size distribution and surface properties of grains, such as wettability, has been of great interest. Rock proxies can be used for petrophysical and geomechanical characterization of rock and assessment of fluid flow and reactive transport in porous media in a more efficient and cost effective approach than using natural rock samples [52,53,54]. 

One of the main advantages of 3D printing over other existing techniques is to have a great degree of freedom in the geometry design and variation in cross-sectional geometries [55]. Moreover, the surface roughness of the micromodel can be controlled during the printing process. For example, consumer-grade stereolithography (SLA) printers can achieve sub-micrometre surface roughness [56]. The resolution of printing depends on the vertical (Z) resolution or the smallest achievable layer thickness, and the horizontal resolution or the smallest feature size that can be made in the XY plane. Depending on the type of printer used, Z and XY resolutions can vary from 0.15 and 1 µm to 100 s of µm, respectively (e.g., Nanoscibe GmbH [57]).

**Figure 4:**
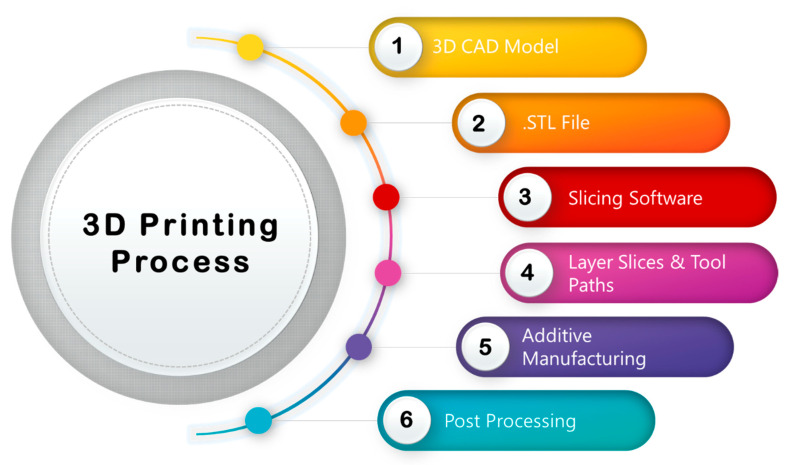
Schematic of the process sequence in 3D printing of an object.

The 3D printing process consists of three main steps, namely modelling, printing and post processing, as shown in Figure 4. Using a Computer Aided Design (CAD) package, a 3D model can be created and exported as a .STL (Standard Tessellation Language) file that is then converted into a series of thin layers using a slicing software to create the 3D object.

Many different AM processes are now available for 3D printing. In some methods, such as selective laser melting (SLM), selective laser sintering (SLS), and fused deposition modelling (FDM), the printing materials are melt or soften to produce the successive layers. Other AM techniques, e.g., SLA and laminated object manufacturing (LOM), cure liquid materials to produce the layers. Further details about different printing techniques have been reported elsewhere [50].

Kitson et al. [58] fabricated microfluidic devices for chemical synthesis using 3D printing techniques. They used a 3D printer (3DTouch^TM^) with the Z resolution of 0.125 mm which allowed them to print channels with approximately circular cross section and diameter of 0.8 mm. They made micro- and milli-scale devices and found 3D printing a very flexible technique to fabricate different designs for mixing points, inlets and outlets and to optimize them in relatively short amount of time.

Watson et al. [55] investigated the application of 3D printing for studying pore-scale fluid flow and transport processes. They used a Formlabs Form 2 stereolithography printer with resolution of 0.025, 0.050, and 0.100 mm in the X, Y, and Z directions, respectively. However, they observed 0.4 mm discrepancy in the channels’ depth between designed and printed models, which can be due to printing orientation. They reported their 3D printed micromodel presented sharper channel edges, more uniform channel width and less surface roughness that those of a similar micromodel fabricated using PMMA. They also performed single-phase tracer tests on both models and compared the experimental results against direct numerical simulations. A better agreement was found between results of PMMA experiment and numerical simulation, which could be due to abovementioned differences in the micromodels [55]. Dimou et al. [59] also evaluated the capability of a FormLab Form 2 stereolithography printer and repeatability of printing process for fabricating pore structures with resolution of 200–500 µm.

In recent years, 3D printed models at different scales have gained more attention in geoscience and petroleum engineering to investigate fluid flow in porous media at the Darcy scale and study macroscopic properties. Further, 3D printing allows us to replicate very similar rock samples (rock proxies) and perform different experiments. Head and Vanorio [60] created 3D printing of rock proxies using photo-reactive resin and based on acquired 3D micro CT imaging to investigate the effect of rock microstructure on macroscopic properties, such as porosity and permeability. Suzuki et al. [61] studied flow mechanisms in fractured systems using 3D printed rock proxies in order to have control over fracture properties. They utilized an X-ray CT scanner (Section 3.2.1) to investigate the accuracy of 3D printed samples. They found 3D printed rock proxies with fracture networks to be very helpful for performing experiments and using experimental results for validating mathematical models. Recently, Ahkami et al. [62] fabricated a 2D fractured porous medium using 3D printing. They employed a new high-resolution PIV method (Section 3.1.4) in order to investigate fluid flow behavior within the pores, open and dead-ended fractures simultaneously.

Ishutov et al. [53] used resin-based 3D printing methods to explore the effect of processing techniques to reduce the observed porosity discrepancies. They showed that using SLA 3D printing can improve the accuracy of the rock proxies’ geometries with a minimum pore size of 390 μm. Moreover, post processing, such as pressurized flushing with ethanol and air, significantly enhanced the accuracy of pore space replication, where porosity difference between the printed and natural samples was reduced from more than 50% to only ~1%. Ishutov et al. [54] concluded that rock proxies can be used for analysis of porous sedimentary rocks, including flow and transport applications. 

### 2.3. Glass-Based Micromodels

#### 2.3.1. Glass-Beads

These models are constructed by filling in a glass container (box or tube) with glass beads [63]. The container can also be a Hele-Shaw cell. Such a cell consists of two glass or acrylic (PMMA) plates separated by a thin gasket to create a gap (typically less than 1 mm). This gap can act as a channel, in which the motion of a fluid (or fluids) takes places, or it can be filled in with small glass beads or spheres. The flow through the gap between two parallel plates is mathematically analogous to two-dimensional flow in a porous medium [64,65].

**Figure 5:**
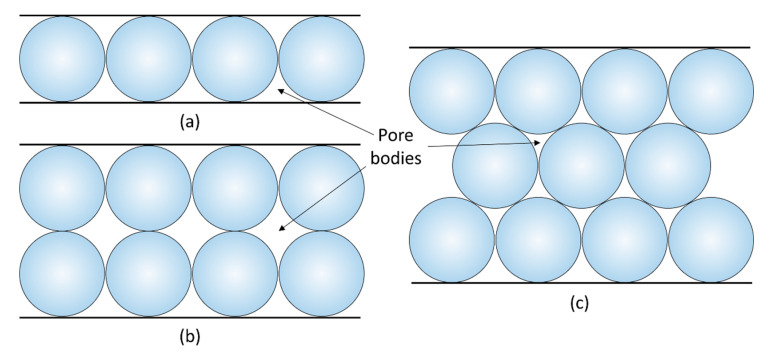
Cross-section of pores in the glass-bead models containing: (**a**) single layer of identical spheres, (**b**) multi-layer cubical packaging of identical beads, and (**c**) multi-layer hexagonal packaging of identical beads.

The inlet and outlet holes in the glass-bead models are typically placed near the cover plate edges or at the sides. However, the models with an inlet in the centre of the cover plate have also been used in some experiments [66,67,68,69]. Glass beads may have different diameters, starting from a few tens of microns. Using beads of a given diameter it is relatively easy to build models of a regular network. This is normally achieved by vibrating the glass container while inserting the beads. The use of beads of different diameters results in the formation of partially-regular and irregular network structures. The shape of pore bodies in the structure is quite limited because it depends only on the diameter of beads and their arrangement, as shown in Figure 5.

Although the construction of glass-bead models is not complicated, because it does not require any sophisticated tools, there are a couple of challenges associated with their construction and usage. The first difficulty is to ensure that glass beads are in close contact with the container walls. If this is not achieved, an injected fluid may flow only through the gaps instead of through pores inside the model. The second drawback is the optical visualization of fluid flow inside pores, in particular when the beads have a large diameter or when the model is three-dimensional, i.e., it has more than one layer of beads, as shown in Figure 5b,c. In such arrangements of glass beads, the depth of focus of a camera zoom lens can be too short to capture a sharp image inside the structure. Optical access to the pores may be additionally obstructed when the plates are too thick. The visualization of flow and transport experiments in glass bead micromodels can be improved by matching refractive index between the beads and the fluid which is known as refractive-index matching (RIM) method [70,71,72]. Rashidi et al. [70] used a refractive index-matched fluid seeded with fluorescent tracer particles and a pulse of solute dye to improve the dynamic measurements of flow velocity and solute concentration within the pore spaces in 3D.

The first glass-bead models were constructed in the early 1950s by Chatenever and Calhoun [73]. These models were based on a Hele–Shaw cell and were used for the investigation of the motion of two immiscible fluids (water and filtered crude oil) in porous structures. The authors designed two flow cells of different sizes and shapes, in which the gap between the two glass plates was filled in with a single layer of glass spheres (φ ≈ 0.18 mm). To encase the glass beads, they used compression covers and gaskets. Flow cells containing more than one layer of glass spheres were also constructed, but problems were reported with the observation of flow phenomena and with the distinction of phases.

An interesting construction of the glass-bead model was presented by Corapcioglu and Fedirchuk [74] who enclosed a single layer of glass beads between two Pyrex plates without the use of any gasket and adhesive. This was achieved by etching of a recess of a certain depth in both plates, placing the glass beads between these two recesses, and applying a thermal process for bonding the plates and glass beads together. The constructed model was used as a tool to observe the flow of a dyed solute through a water-saturated porous structure. The dye was used for enhancing the contrast between the two fluids. Nowadays different dyes are widely used, in particular in multi-fluid flow experiments. 

In general, the fabrication process of glass-bead models is very mature and not complicated. Although these types of models are suitable for high-pressure applications, as demonstrated for instance by Wang [75], they also present a few drawbacks, as listed in Table 1. The main disadvantage is a limited number of pore network patterns than can be achieved with glass beads. This means that these models are not suitable for the replication of internal structures of real geomaterials because the structures of rocks contain many irregularities that cannot be reproduced with spheres only.

#### 2.3.2. Glass Wafers/Plates

The properties of glass, such as high transparency, hardness, chemical resistance, chemical inertness and thermal stability, make this material a preferred substrate over silicon or polymers for the manufacturing of microfluidic devices for geological and petroleum engineering research. The most common techniques used for the generation of flow patterns on glass substrates are reactive ion etching [76,77,78,79,80,81] and wet (chemical) etching [80,81,82,83,84]. Other techniques, such as direct laser writing [79,85,86,87,88,89], selective laser-induced etching (SLE) [90,91,92,93], and direct laser writing and laser micro-welding [94,95], are less common, however they are very attractive for the rapid prototyping of microfluidic devices. Moreover, the SLE process enables the generation of complex three-dimensional structures inside glass, as highlighted in Table 1, and this eliminates additional fabrication steps related to the bonding of two glass plates together.

Reactive ion etching (RIE) is a ‘dry etching’ technology in which a material is selectively removed from the material surface by a chemically reactive plasma that is generated under low pressure (<13 Pa or 0.13 mbar) by a strong radio frequency (RF) electromagnetic field. Gases, such as SF_6_, C_4_F_8_, CF_4_, and CHF_3_, are typically used for etching glass substrates [79,80,81]. Additional gases such as H_2_, O_2_, He, and Ar, are often used to improve the etching process, e.g., to increase the etching depth or to reduce the roughness of the etched surfaces. Since the RIE process is anisotropic (directional), this enables the generation of channels (pores and throats) with almost vertical sidewalls (the angles up to 88°). With the RIE process, it is feasible to generate channels with depths of up to 430 μm, surface roughness (Ra) < 10 nm, and an aspect ratio (depth to width) of up to 40 [79]. Since the process is relatively slow (etching rate < 0.0167 μm/s or 1 μm/min) and the plasma generation can be easily stopped (simply by switching the generator off), the depth of etched structures can be controlled with high accuracy. Low etch selectivity of RIE (typically < 5:1) means that this method is most suitable for the generation of relatively shallow structures. If structures deeper than a few tens of micrometres are required, it is necessary to use thick masks, and this affects the surface roughness and the spatial resolution of etched surface features. Photoresist SU-8, bulk silicon, as well as metals (such as Cr and Ni) are used as masking layers [80,81]. The photoresist masks are manufactured by photolithography (more details in Section 2.1), the silicon masks by a combination of photolithography and etching, whilst the metal masks by using both photolithography and electroplating [79].

The other method commonly used for the generation of microfluidic patterns on glass is wet etching. This process involves using liquid chemicals (so called etchants) to remove material. Unlike RIE, the wet etching process is isotropic, i.e., the etching rate is equal in all directions. Glass substrates are typically etched by using highly concentrated hydrofluoric acid (HF). Solutions combining HF with other strong acids, such as HCl, HNO_3_, H_2_SO_4_, and H_3_PO_4_, are also used, mainly to enhance the etching rate.

Mask layer quality can be an issue in the generation of pore network patterns on glass by wet etching. As noted by Iliescu et al. [80,83], the masks should be hydrophobic and free of any cracks. If the masking layer is hydrophilic, however, the etchant can penetrate through cracks and generate pinholes and notch defects on the glass substrate. As with RIE, photoresist and silicon, as well as silicon carbon (SiC) and metals (such as Cr, Au, Cu, and Ti) are used as masking materials. The choice of the masking material depends on the required depth and quality of the etched features. A photoresist mask is limited to shallow etching (depth < 25 μm and etching time < 180 s) [83]. Silicon-based masks, which are fabricated by either plasma-enhanced chemical vapour deposition (PECVD) or low-pressure chemical vapour deposition (LPCVD), enable generation of deep features (up to 250 μm) on glass. The metal masks are typically made of Cr and Au or Cr and Cu, where the Cr layer is used to improve adhesion of gold/copper to glass. These masks allow borosilicate glass substrates to be etched up to depths of 100 μm [82]. Deeper structures (up to 500 μm) are achievable by using multilayer masks containing a combination of metals and hard-baked photoresist [83]. The presence of photoresist is essential due to its hydrophobic nature to make more difficult penetration of the etchant through any small defects in the masking layer [80]. 

The advantages of using a wet etching process are the high etching rate (approximately 7 μm/min for borosilicate glass), high selectivity (up to 30:1), as well as low roughness of etched surfaces (even 10 nm) [83]. However, its isotropy means that it is limited to low aspect ratio channels (approximately unity). Such channels contain walls with rounded corners, undercuts, and so called “notch defects” which often are generated on glass under a masking layer [82]. Also, the chemical and disposal costs can be very high because, during wet etching, the etching material must be covered entirely with etching solution and this solution must be changed on regular basis in order to maintain the same nominal etching rate. 

Pore network patterns generated on glass substrates form rigid microfluidic systems which are characterized by high transparency, thermal stability and chemical resistance, as highlighted in Table 1. It is important that these properties are not changed by the subsequent bonding process. Thermal bonding (also called ‘fusion bonding’) is the most common technique used for bonding two glass plates together [80,81]. This technique involves three major steps: (i) cleaning, (ii) pre-bonding, i.e., bringing two glass plates to optical contact by pressing them together, and (iii) thermal treatment of the pre-bonded glass plates by placing them in a furnace and keeping at a high temperature (near the annealing point) for several hours. Very clean and flat surfaces are essential to achieve a successful bond between the two glass plates. The presence of Newton’s rings following the pre-bonding process indicates that the cleaning of glass plates was insufficient and the process must be repeated. The two glass plates must also have identical or at least very similar thermal properties [81]. The small coefficient of thermal expansion of fused silica (CTE ≈ 0.6 ppm/K) and Borofloat^®^33 glass (CTE ≈ 3.3 ppm/K) makes them resistant to thermal shock and thermal stress, and hence they are suitable for thermal bonding. Because Borofloat^®^33 glass contains a high concentration of alkali ions (approximately 4% by weight NaO_2_), it can also be bonded to Polymers such as poly-di-methyl-siloxane (PDMS) following the oxygen plasma treatment, as described in Section 2.2.1, or to silicon by anodic bonding (see Section 2.4 for details).

Glass-based microfluidic devices are able to withstand very high internal pressures up to a few tens of MPa (several hundred bars) if they are appropriately designed [96,97]. Therefore, it is unsurprising that these devices have found use as physical representations of pore network models in geological and petroleum engineering research. They have been used for investigation of many different fluid transport processes in porous media [98,99,100,101,102,103,104,105,106,107,108,109,110,111,112,113,114,115,116,117,118].

### 2.4. Silicon-Based Models

Pore network structures can also be generated on the surface of silicon wafers, using essentially the same fabrication techniques as for the generation of pore network patterns on glass [80]. Because silicon has different physical properties than glass, the process parameters must be properly adjusted, but the process principles remain unchanged. Since silicon is opaque in the visible, direct optical visualization of fluid flow processes inside pore network structures is possible only at the surface of a silicon wafer bonded to a transparent (typically glass) substrate. 

The main advantage of using silicon over glass substrates is the ability to generate pore network structures with very high (sub-nanometre) resolution and accuracy. The etching techniques that have been adapted from the semiconductor (micro-electronics) industry enable the generation of pores and throats whose size is comparable to that of pores and throats in real rocks [18,119,120,121]. 

The ‘Bosch’ process is the most common dry etching technique used routinely for the manufacture of microfluidic devices from silicon wafers [80,81], although other techniques (e.g., cryogenic plasma etching) are also used [122]. This process involves two steps, namely passivation and etching, which are up to the point where a desired depth of the structure is achieved. In the passivation step, a thin conformal layer is applied onto an exposed silicon surface by generating C4F8-based plasma. In the next step, this protective layer is removed by ion bombardment, and then the silicon wafer is isotopically etched for a very short period of time (a few seconds). The etching process uses sulphur hexafluoride (SF_6_). By repeating the passivation and etching steps, the Bosch process enables the generation of very deep channels with nearly vertical sidewalls.

Wet etching methods are also used for the generation of pore network structures on silicon wafers. Depending on the etch solution used, the etching can be isotropic or anisotropic. Isotropic etching is obtained by using HNA (hydrofluoric, nitric, acetic) solutions [80]. The etching rates up to 90 μm/min can be achieved with these solutions. Anisotropic etching, in turn, is possible by using aqueous KOH (potassium hydroxide) solution which is less aggressive than the HNA solutions. For instance, the 40% KOH solution enables the etching rates to be achieved between 0.03 and 2 μm/min, depending on the crystallographic plane of silicon [81]. 

Anodic bonding is the most common method used for bonding silicon to glass without using adhesives. Unfortunately, this is not suitable for all glass materials. Generally, it is essential to use glass containing NaO_2_ compounds (e.g., borosilicate glass) because under the high electric field and temperature the positive sodium ions (Na^+^) become mobile and migrate towards the cathode, whereas the negative oxygen ions (O_2_^−^) drift towards silicon, occupying a depletion region at the silicon/glass interface. This leads to the formation of a strong and stable bond with the silicon surface that can withstand very high pressures [80,81]. Silicon-based micromodels have been widely used to study transport processes in porous media [18,119,120,121,123,124,125].

### 2.5. Geomaterial-Based Models 

The level of complexity in pore structures of micromodels has increased significantly and micromodels with more realistic pore structures mimicking real rock samples have been fabricated. However, the materials used for fabricating micromodels may not be able to fully capture the subsurface fluid and rock interactions, e.g., surface roughness, wettability, and reactive flow [126]. Moreover, due to 2D (or 2.5D for depth-variable patterns) nature of micromodels, the connectivity of corners and crevices in real rock samples are not properly replicated which can affect flow and trapping mechanisms happening in multiphase flow experiments [127]. The improvement in fabrication techniques for producing more realistic micromodels can be divided into three main categories: wettability alteration of micromodel material; engraving a pore network on a crystal or a section of rock sample (geomaterial) and; enclosing it with a transparent plate, and finally coating the micromodel surface with minerals (geomaterials). 

Partially wetted materials and techniques to alter micromodel surface wettability have been used to imitate non-uniform wettability conditions in real rocks. Coating a water-wet surface of e.g., glass substrates with octadecyltrichlorosilane (OTS) solution alters the wettability toward non-water wet. Chang et al. [128] employed a four-steps treatment process to produce two mixed-wet wettability patterns in 2.5D silica micromodels. Their treatment process incudes (1) cleaning with acetone, drying by air and saturating with ethylene glycol, (2) injecting surface coating OTS/hexane solution, (3) injecting hexane to remove excess OTS, and (4) air drying and curing in oven at 100 °C. Hu et al. [104] altered the wettability of a pore network structure etched on fused silica plate in a micromodel by using a coating solution consisting of a mixture of diluting Aquaphobe-CM (PP1-AQCM, Gelest) and hexane (ACS grade, EMD). They injected the coating solution into the micromodel and left it for several hours to allow the coating solution to react sufficiently long with the pore network surfaces.

Murison et al. [129] and Hiller et al. [130] created glass bead packs with heterogeneous wettability using two gold and chlorotrimethoxysilane (CTMS) for coating and utilized X-ray CT imaging (Section 3.2.1) to establish a link between capillary pressure-saturation curves and pore-scale wetting heterogeneities.

Schneider et al. [30,31] fabricated PDMS micromodels with well-controlled wettability patterns where they reproduced wettability heterogeneity, which is observed in many hydrocarbon reservoirs. Wettability alteration was done by UV-initiated graft polymerization of poly acrylic acid (PAA). Lee et al. [131] adapted the stop-flow-lithography (SFL) technique to produce micromodels with cylindrical pillars with specific wettability properties (water-wet, oil-wet and intermediate) in different regions of the pore structure. They visualised the effect of wettability heterogeneity in flow through experiments, where water displaced decane or vice versa.

Other fabrication techniques have been proposed to include geomaterials in the fabrication process of micromodels to produce a more realistic representation of natural rocks. Song et al [132] developed a multi-step method to fabricate a micromodel with channels etched into a natural calcite crystal. They sectioned a large calcite crystal into 3 mm thick wafers, that were then coated with a thin layer of beeswax. The pattern of interest was inscribed through the wax using a laser cutter, and the wafers were then immersed in 10% hydrochloric acid (HCl) to dissolve the calcite and create the pattern. Finally, they removed the wax, drilled two holes as inlet and outlet and applied a thin layer of Scotch-Weld Instant Adhesive CA40 to a borosilicate glass plate to close the pattern. They studied geochemical reactions and pore-scale fluid–rock interactions in real time in two micromodels, namely a single straight channel and a square matrix pattern [132]. 

Porter et al. [126] created micromodels by etching pore-scale fracture patterns taken from 3D computer microtomography scans of shales into thin sections of shale sandstone and siltstone. They used a custom-built femtosecond laser direct-write system to etch the pattern. A glass plate was used to seal the top of each etched pattern. They spread a thin and yet uniform-thickness layer of UV epoxy over the glass plate and cured it in two steps. The first and partial curing step was to avoid spreading of the epoxy into the etched porous pattern and the second, but complete curing step was after clamping the glass plate to the etched substrate. It is worth mentioning that they also used thin (25 μm) pressure sensitive adhesives to attach the glass plate to the etched substrate. Using real shale samples ensured that the physical and chemical properties of the models are realistic, but it could not overcome the inherent heterogeneity between shale samples. They visualized fracture–matrix interactions during imbibition, compared the displacement of water by supercritical CO_2_ in straight fractures etched in glass and shale, and showed water displacement by supercritical CO_2_ in a real fracture pattern. Gerami et al. [133] presented a similar work, where they applied a laser etching technique (three-dimensional laser micromachining) to produce a fracture pattern into a coal surface. X-ray micro-CT imaging was used to obtain a pattern of the microfractures and cleat structure of coal. Based on wettability and surface roughness measurements of coal samples, they concluded that these two properties are highly heterogeneous and using a geomaterial micromodel is the only fitting approach to capture them in pore-scale studies. 

Zhu and Papadopoulos [134] utilized a microscopic cylindrical packed bed to perform primary drainage displacement. They used PYREX^®^ melting-point tubes (1.5–1.8 mm outside diameter OD) and through a process of heating and pulling, they created channels with internal diameter of around 90 μm at the middle of the tubes. Cryolite particles, a few times smaller than the channel’s inside diameter (ID), were used throughout a multi-step process to fill the channels. Soulaine et al. [135] embedded a calcite crystal at the centre of a straight PDMS microchannel (1.5 mm × 0.2 mm cross-section) to study the dissolution process and validate their numerical simulations. Bowden et al. [136] developed a technique for rapid and cost-effective fabrication of micromodel with unconsolidated beds of mineral grains packed into channels etched in soda lime glass. They used soda line glass spheres to represent silica; however, for other minerals they crushed rock or crystals. Tanino et al. [137,138] used a similar micromodel with quasi-monolayer of crushed marble packed inside a channel to study mixed-wet conditions on oil recovery and film flow and piston-like displacement in oil/water two-phase flow. They used a variable wavelength light source and a high-speed 24-bit colour camera coupled with an optical microscope to image the depth-integrated fluid distribution. As part of their study, they compared the experimental results obtained from micromodel testing and core flooding to understand the effect of advancing contact angle on residual oil saturation. Singh et al. [139] embedded a real rock thin section inside a PDMS channel and called their micromodel a real rock-microfluidic flow cell (RR-MFC). Their objective was the direct visualization of flow and transport in the pore space of a real rock sample. A 500-μm thick sandstone section from a reservoir of the North Sea was mounted inside a micromodel as its porous structure. They used different microscopy techniques to determine the mineralogy, geochemistry, and rock pore networks, and characterize reactive multiphase flow.

Coating the surface of micromodels with a certain mineral is an interesting approach in order to replicate surface roughness, wettability and chemistry of natural rocks. Song and Kovscek [140] presented a method to coat a silicon micromodel surface with clay particles of kaolinite. They described a detailed procedure of coating in their work. The agreement between the structural characterization of deposited kaolinite particles and Berea sandstones validated the applied deposition method. Using the fabricated micromodels, they investigated the effect of mixed wettability conditions on hydrocarbon recovery and improved oil recovery by low salinity waterflooding. Song and Kovscek [141] investigated the behaviour of low salinity brine injection in clay-rich sandstones using their clay-functionalized etched-silicon micromodels.

Lee et al. [142] developed a technique for in situ growth of calcite (CaCO_3_) on the micromodel surface made of glass in the controlled area. They fabricated micromodels with calcite pillars and numerous chemical surface modifications were performed to achieve in-situ growth of calcite. Following on this work, Wang et al. [143] introduced a method to coat glass micromodel channels with nanocrystals of calcite (CaCO_3_) in order to resemble a carbonate system for water/oil displacement experiments. After multiple chemical surface modifications for growing calcite nanocrystals on the glass surface, they tuned the wettability of calcite layer through an aging process so the fabricated micromodels mimic carbonate rocks. Recently, Alzahid et al. [127] presented a relatively simple and fast process to functionalize the pore space of PDMS micromodels with selective rock minerals. They used quartz and kaolinite as sandstone representatives, and calcite to represent carbonate. After fabricating a PDMS slice with a desired porous structure, they placed the selected mineral solution on top of that. They air-dried and cleaned the slice and assured there were no mineral particles on the top of pillars in porous structure which may cause problems in bonding to the blank cover PDMS slice. Finally, they performed a plasma treatment after placing a blank PDMS slice on the top of PDMS slice with mineral coated porous structure. To alter the surface wettability of PDMS, that is naturally oil-wet, they used a technique developed by Trantidou et al. [144] to combine oxygen plasma and polyvinyl alcohol (PVA) treatments.

Ishutov et al. [52] attempted to reproduce Fontainebleau sandstone samples combining micro CT imaging and powder (gypsum)-based 3D printing. The resolution of their 3-D printers could not produce pores or grains less than 150 µm in diameter. Due to the limitation in resolution of their used printer, they had to scale up the rock pore system by different magnification factors of 5, 10 and 15. Although permeability and mean pore throat radius of the 3D printed proxies matched with literature data (laboratory measurements) and digital rock analysis, they observed discrepancies in porosity values. Therefore, they found it a real challenge to replicate pores of natural samples. Kong et al. [145] studied post-processing effects on 3D printed microstructure characteristics. They utilized 3D printing with gypsum powder to fabricate four cylindrical rock proxies without any designed porosity. They then applied different types of infiltrants and coating conditions after the printing process and showed that infiltrants mainly affected the distribution of nanopores, whereas coating did not have any significant impact on the pore structure. 

Recently, the same group used 3D printed (gypsum powder) rock proxies to validate upscaling methods, which are used to model mechanical properties of a rock type from micro to macroscale [146]. They measured geo-mechanical properties (e.g., Young’s modulus, Poisson’s ratio and volume fraction of each mineral phase) for 4-mm size fragments of crushed 3D printed rock proxies and used them as input parameters to Mori–Tanaka scheme, self-consistent scheme method, and differential effective medium (DEM) theories to estimate Young’s modulus at macroscale. To verify model predictions and compare the performance of each upscaling method, they performed triaxial compression tests on two similar 3D printed core samples (length and diameter of 57.15 and 38.1 mm respectively) and acquired modulus values at macroscale. They concluded that the application of 3D printing for rock mechanics studies needs further improvements in different aspects of printing from materials and printable features to post-processing. 

## 3. Imaging Techniques

Imaging techniques are widely used to view and understand important processes in physics, biology and engineering and reduce the cost and time required for acquiring data and information [147]. Optical (visible light) imaging can be used to noninvasively view inside an object and obtain detailed images of its inner structure. Non-optical wavelengths are also used to view additional structural properties, or to provide an image through a material that is opaque in the visible spectrum. In geoscience and geo-engineering, imaging techniques allow direct observation of pore characteristics (size, shape, structure, connectivity, and distribution) and fluid flow and transport mechanisms in porous media. The resultant images enable qualitative and quantitative analysis and the validation of mathematical models. 

The following sections analyse different imaging techniques used for pore-scale characterization, fluid flow and transport in homogeneous, heterogeneous, and fractured porous media. Table 2 summarizes and compares all the different techniques that are reviewed here. The comparison includes the maximum possible dimensions of the acquired images, field of view (FOV), or image resolution (IR), applications, advantages, and limitations of each technique in general, and specifically in the area of geosciences, petroleum, and geo-engineering.

### 3.1. Optical Imaging

Optical methods have been employed extensively for studying multiphase flow in microfluidic devices [5]. Although the main application of optical imaging of micromodels is qualitative analysis (front evolution and flow, fluid configuration and governing mechanisms of processes), image analysis can also provide valuable quantitative information which can be used for verification of mathematical models. Average and local fluid saturations in a micromodel can be estimated using image analysis. According to the parameters of interest, micromodel patterns, and type of fluids and compositions, different imaging resolutions and colour depths are required. A high acquisition rate is crucial for the visualization of dynamic experiments where fluid distribution needs to be recorded as a function of time [8].

Table 2 summarises the optical imaging methods commonly used for visualization of fluid distribution, two-phase flow, solute transport, particle dynamic transport and reactive transport in micromodels. They include camera, microscope-camera, photoluminescent volumetric method, confocal microscopy, Raman microscopy, and micro particle image velocimetry (µPIV) [18,73,112,114,115,116,117,139,149,150,151,152,153,154,155,156,157]. Generally, these methods are low cost and probably represent the easiest option for imaging micromodels [5]. However, for studying some processes at high-pressure and high-temperature conditions, specially designed experimental set-ups including high-pressure vessels with sapphire glass windows are required [112,115,116,158].

#### 3.1.1. Camera & Microscope–Camera

High resolution cameras have been used for acquiring images and videos of micromodel experiments. An extra objective lens can be used to increase magnification. High-resolution digital cameras with charge coupled device (CCD) and complementary metal-oxide semiconductor (CMOS) sensors have been successfully used for monitoring micro-scale phenomena. The number of frames per second (frame rate), resolution, and colour depth of cameras has significantly improved in recent years and hence satisfy the requirements for imaging at micromodel scale. High resolution cameras have been employed to visualize saturation changes and flow mechanisms of different enhanced oil recovery processes, CO_2_ storage, solute transport, and multiphase flow in micromodel experiments [7,112,114,115,116,117,156,159,160]. 

Typically, a micromodel is placed under a microscope lens and a camera is mounted on the microscope’s ocular [18,73]. The microscope-camera set up is used when high resolution imaging (e.g., 1 to 2 μm) is required. The FOV is limited in this method and unlike camera-only imaging, dynamic monitoring of whole micromodel is challenging as either micromodel or ocular of the microscope should move rapidly which could have negative effects on the experiment and image quality. This method has been used for visualizing flow in 2D flow cells and micromodels to study interfacial area and transport in porous media, but it is not suitable for 3D models [9,18,73,161].

#### 3.1.2. Fluorescent and Confocal Microscopy

Photoluminescent volumetric imaging (PVI) was introduced by Montemagno and Gray [149] and used for quantitative study of dynamic changes in distribution of phases and interfaces in a 3D porous media replica. A very high resolution of 1 µm for a sample volume of 10 mm^3^ can be obtained using this technique. The imaging system mainly consists of a laser, a lens and a CCD camera. Active fluorophores are dissolved in the fluids and excited by the laser beam and as a result the fluid–fluid interfaces are illuminated and captured by a CCD camera. Refractive index matching between fluids and solid materials is required when using this method. All 2D images captured should be processed to obtain a 3D image of porous structure and fluids distribution. Stöhr et al. [151] employed this method to study the dispersion of a tracer dye in single-phase flow and the imbibition process of waterflooding in a 3D flow cell. Interface mass transfer, nonaqueous phase distribution and in-situ bacterial processes can be studied using this imaging technique [151].

Confocal microscopy has a similar basis to laser induced fluorescence technique, but it is a point-by-point visualization method. When the process of interest is relatively slow or very high resolution (submicron) is required, confocal microscopy is an ideal imaging method. The effective depth is up to 250 µm in this method. A 3D image of a sample can be created using 2D images obtained at sequential layers. Grate et al. [157] used this technique to study the distribution of non-wetting fluids and wetting film structure in a 2D micromodel. 

#### 3.1.3. Raman Microscopy

The Raman effect was first discovered by Chandrasekhara Venkata Raman in 1928 and published by Raman and Krishnan in Nature [162]. A molecule scatters incident light from a laser light source in which most of scattered light has the same wavelength as the light source. However, a small amount of light, typically 0.0000001%, is scattered at different wavelengths, which is called Raman Scatter. Depending on the chemical structure of the sample, the Raman scattering results in a unique spectrum. Raman spectroscopy is a non-destructive technique which can be used for microscopic analysis, with a sub-micron spatial resolution (0.5–1 µm). It requires small sample volumes with little or no preparation but provides detailed information about chemical structure, phase and polymorphy, crystallinity and molecular interactions. Raman spectroscopy can be used for analysis of a multilayered sample or samples beneath the surface of a transparent container (e.g., glass or plastic) and is ideal for examining aqueous solutions. A Raman microscope combine a Raman spectrometer and a standard optical microscope to allow for high magnification and high resolution [163,164,165]. 

Raman spectroscopy has been successfully employed for in-situ mineralogical characterization in micromodels [139,152,153,166]. Applications and challenges of integrating Raman systems with microfluidics was reviewed by Chrimes et al. [152]. Both Raman systems and microfluidics works with small sample volumes which makes them compatible to be coupled. A large amount of research in pharmacology, forensics and bio-analytics have been already generated using these technologies. The applications of this integration have also gained interest in the field of geoscience, and in particular mineral characterization and reactive transport.

Raman measurements have shown very promising in assessment of the accessible reactive surface area and reaction rates for describing and modelling mineral precipitation and dissolution in porous media.

#### 3.1.4. Micro Particle Image Velocimetry (µPIV)

Particle image velocimetry (PIV) is a well-established optical and nonintrusive technique for macroscopic fluid and granular flow measurements [167,168,169,170]. The fluid is seeded with sufficiently small particles which are assumed to follow the flow. The system is illuminated and since particles are visible, it is possible to record particles’ motion and to calculate speed and direction of the flow being studied. As a result, a 2D or even 3D velocity field can be obtained by PIV. Micro particle image velocimetry (µPIV) is a modified version of PIV which can achieve spatial resolutions in the order of micrometres. µPIV is used to investigate the flow in microfluidic devices and to measure flow-field properties at micron scale [154]. The µPIV measurements with detailed information at micron scale can also serve as a reliable reference to pore-scale numerical simulation for validation and improvement [171].

Santiago et al. [172] developed a µPIV system to measure instantaneous and ensemble-averaged flow fields in microfluidic devices. Their system consisted of an epifluorescence microscope, a CCD camera, and an external light source. They used fluorescent polystyrene beads with a density matched to the experiment fluid. 

The selection of right seed particle size is significantly important in µPIV, as seed particles must be small enough to smoothly flow with the bulk fluid without disrupting the flow and blocking the channels. At the same time, particles must be large enough to dampen the effects of Brownian motion that can cause random error in the measurement of the particle displacement between images and this error can be very significant for slow flows.

As the seeding particles in µPIV are small compared to the wavelength of the illuminating light, either fluorescent imaging or efficient image acquisition and processing [171,173] are required. The quality of µPIV measurements and the resolution of µPIV systems have been improved through further developments in measurement method, interrogation techniques and filtering schemes [174,175,176]. Perrin et al. [177] used µPIV to study the flow of Newtonian and non-Newtonian fluids in a silicon-based micromodel using a Berea sandstone pore pattern. They reported the importance of particle selection in order to avoid any chemical reaction and seed aggregation. 

The application of µPIV in flow experiments gets more challenging when there are two or more immiscible fluids in the system. Blois et al. [178] discussed the associated challenges with imaging flow of two immiscible fluids and described an imaging method which couples refractive index matching and fluorescent signal separation to overcome these challenges. They used PDMS micromodels with a regular pattern for flow experiments and a dual-camera µPIV setup for visualization. They performed a drainage test and observed front evolution through preferential pathways. The images showed that the front is unstable with abrupt changes in velocity magnitude and direction which are prone to cause Haines jumps. Moreover, they suggested using high-speed image acquisition to capture these short time events. Heshmati and Piri [179] developed a new two-phase and two-fields-of-view μPIV experimental apparatus to investigate velocity fields, shear stress at the fluid/fluid interfaces and the trapping and reconnection mechanisms at two-phase flow conditions in PDMS micromodels. Study of fluid movement in certain pores and across the entire micromodel was possible due to integration of small and large FOV modules with the apparatus. Most recently and for the first time, Roman et al. [173] investigated the magnitude of the interfacial momentum transfer force for different flow conditions using a μPIV experimental set-up.

### 3.2. Tomography Techniques

Tomography is a non-destructive radiologic imaging technique using a penetrating wave, such as X-rays. In tomography, high-resolution images of internal structures of an object are obtained by focusing on a specific plane within the body of that object. The main drawback of conventional radiologic images is the loss of information in one dimension. Radiography puts information of a 3D object into a 2D image which can be misleading [14]. The simplest method is linear tomography, in which an X-ray source and an X-ray sensitive film or sensor move in a straight line during the exposure, and by applying different direction and extent of the movement, different focal planes which contain different structures of interest can be selected. Focal plane tomography remained the conventional form of tomography until the early 1970s, when computerized or computed tomography (CT) was introduced [180]. Since then, CT has been used extensively for different applications, including geoscience research [181]. 

In the following subsections, tomography-based imaging techniques that have been used for characterizing pore structure and understanding pore scale processes in porous media are reviewed. 

#### 3.2.1. X-ray Computed Tomography (X-ray CT)/X-ray Micro- Computed Tomography (X-ray µCT)

**Figure 6:**
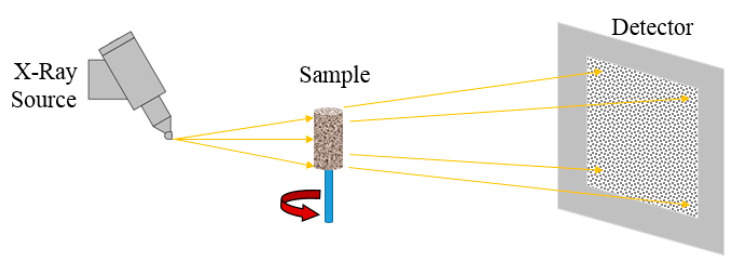
A typical X-ray CT set up with a cone beam configuration. In this configuration the distance between X-ray source and sample can be very small (≈1 mm).

Unlike the traditional radiography which only one 2D-image is taken from an object from a certain angle, in Computed Tomography (CT) lots of radiographs are taken from an object from different angles as an object is rotated. In CT either the X-ray source rotates around a sample or the source is fixed and the sample rotates (Figure 6) and therefore each image is taken from a very slightly different angle. The series of 2D images are mathematically reconstructed into a 3D image via a computer program. In addition to being a non-destructive imaging technique, one real advantage of X-ray CT is to reveal and quantify internal structures of the object by moving through its reconstructed 3D image.

##### X-ray Imaging Techniques

Imaging tomography mainly includes transmission or absorption, phase contrast and fluorescence techniques. The following paragraphs explain these techniques briefly and more details are available in provided references.

The transmission method is based on absorption of an X-ray beam, as it passes through the sample body. Scattering and absorption attenuate the signal when X-rays pass through a sample [182]. The attenuation of X-rays after passing through a solid object is obtained from the Lambert–Beer’s law which is for a pure monochromatic beam (all photons have the same energy and wavelength):

(1) $$I=I_{0}exp\left(-\epsilon x\right),$$

where *I* is the attenuated intensity after the X-rays have passed through the object with a thickness of *x*, *I*_0_ is the incident radiation intensity, and *ϵ* is the linear attenuation coefficient. The X-ray energy, the material’s density and atomic number control the X-ray attenuation [12,183,184,185]. The decrease in the X-ray beam’s intensity is measured with X-ray detector panel and the spatial distribution of attenuation values within the sample is determined from multiple ray measurements using computed tomography process. 

When the X-ray beam traverses the sample, the beam’s phase and amplitude are altered [186]. The phase change (shift) cannot be measured directly by the detector, but the intensity change can be recorded by the detector and the phase change can be estimated through a phase retrieval process [187]. Therefore, the phase contrast technique is based on processing the recorded intensity changes. As the change in beam’s phase per unit path length is larger than the change in the beam’s amplitude, the contrast of images in phase contrast is higher than that in transmission [186]. The phase contrast technique is very applicable for materials with low atomic number elements, which have poor absorption contrast. Geomaterials have lots of constituents, usually with high atomic number and good absorption contrast. As a result, there has been less attention toward application of phase contrast technique in geosciences [12].

X-ray fluorescence tomography (XRF) was proposed by Boisseau [188] to obtain 3D images of trace element distributions. When the energy of an emitted X-ray photon reaches approximately the binding energy of the core level electrons, core electrons are excited and ejected from the atom (i.e., as photoelectrons). For the atom to return to a stable state, electrons from the outer level shell with higher energy decay back to the lower level shell and fluorescence emission is the result of the differential energy between two electron levels [188]. An X-ray pencil beam is used in this technique together with a fluorescence detector to collect X-ray fluorescence. XRF provides detailed information about the material’s elements and their state, although it has a very slow acquisition process and relatively slow 3D volume reconstruction due to the low flux of fluorescence and voxel-based scanning and data collection. XRF image resolution can be in the order of few microns and its penetration depth is just a couple of mm [12]. Micro-XRF has been used in plant and soil sciences, biology, and geosciences either as a standalone technique or in combination with other techniques [189,190,191,192]. 

##### Types of X-ray CT

There are three types of X-ray CT systems: industrial X-ray, medical CT, and synchrotron micro-tomography, depending on the X-ray energy and source, means of sample manipulation and detector geometry. Improvements in data collection and processing have resulted in developing fast lab-based X-ray micro-tomography systems (X-ray µCT). Synchrotron radiation is electromagnetic radiation that is emitted when accelerated charged particles (with an speed close to the speed of light) are forced to change direction by a magnetic field [193,194,195]. Recent advances in synchrotron imaging allow spatial and temporal imaging of dynamic processes such as fluid displacement in porous media [195,196].

The sample sizes for µCT vary and can go up to 40 cm, however in geosciences, typical sample sizes are ranging from 1 mm to 5 cm [14]. Although synchrotron µCT has the best-reported resolution, there is a limitation of sample size, which may cause uncertainty in constructing the bulk image. Wildenschild et al. [184] reviewed medical CT, industrial X-ray, and synchrotron µCT and compared their imaging results. As presented in Table 2, the spatial resolutions of medical CT, industrial X-ray, and synchrotron µCT are around 200–500, 50–100, and 0.3–30 μm, respectively. Industrial X-ray systems with a spatial resolution of 10 μm have been used by Van Geet and Swennen [197] for 3D fracture analysis. They could scan samples with diameter up to 65 mm with voxel size of 70 × 70 × 70 μm^3^, while for smaller samples with 5 mm in diameter they could go down to 10 × 10 × 10 μm^3^ voxel size. Recently, industrial X-ray systems with a much higher spatial resolution of 0.7 μm have been used to study the cracking process of layered shale [198]. Schlüter et al. [199] reviewed techniques for the image enhancement and image segmentation of X-ray µCT data. They concluded that image artefacts and noise can be removed with image processing methods. 

In addition, 4D X-ray CT extends X-ray imaging to assess and visualize dynamic processes, such as multiple phase flow and solute transport in pores structures, with sufficient spatial and temporal resolutions, especially on the scale of milli- to microseconds [200,201,202,203,204]. The result of 4D imaging is a series of uninterrupted 3D images of the internal structure of the material during a dynamic process of interest as a function of time [204,205]. The temporal resolution depends on the duration of one full 360° scan which varies from 30 min to about 10 s. In fast continuous scanning, the X-ray source and detector rotate at temporal resolutions in order of a few seconds every full scan. Extremely fast imaging available at synchrotrons, attains even a sub-second time resolution [204]. Although continuous X-ray exposure must be avoided for strongly attenuating samples (which can cause temperature rise in the sample), for most non-living materials, it is safe to perform 4D imaging. It is worthwhile to mention that the temperature rise in reactive samples can affect the reaction kinetics, e.g., the dissolution rate of minerals [206,207].

The concept of dynamic imaging has been used in the past and in-situ devices such as micromodel visualization and viewing cells have been developed to achieve temporal analysis of different process at pores structures. However, in order to obtain 4D images for some high-speed events, a custom experimental set-up is required [202,204,208]. One of the essential requirements in fluid flow experiments, is the size and composition of the sample holder which confines the sample [208]. In 4D µCT, the sample diameter should be small enough to achieve the desired resolution in microns, considering minimal X-ray source-to-object distance. Moreover, the sample holder should be as transparent as possible to X-rays. A gantry-based 4D µCT system reaching 5 µm spatial- resolutions and 12 s temporal resolutions was jointly developed by X-ray Engineering (XRE) and Ghent University’s Centre for X-ray Tomography (UGCT) [200,201,204]. They investigated several complicated fluid flow and transport processes in complex porous media.

Shastry et al. [208] developed a flow cell which could mimic the dynamic process of their interest, whilst satisfying the imaging requirements. They used 4D µCT to investigate the removal of oil from porous media and applied contrast agents to image low attenuating samples.

##### Applications of X-ray CT

X-ray CT is a non-destructive and non-invasive imaging technique that can be used for imaging and direct observation of properties and processes in tissue, bone, rock, and metal. Therefore, it has a very wide range of applications, including medical science, biology, earth science, material science and many other areas [209,210,211]. Characterization of pore shape and structure [12,212,213,214], saturation distribution [200,215,216,217,218,219], fluid flow mechanisms, soil deformation [220], and reactive transport [221] have been some of the main applications of X-ray CT in geological and hydrological studies. 

Shah et al. [222] investigated the impact of voxel resolution in X-ray µCT imaging on the prediction of petrophysical properties of rock samples. They scanned the same physical FOV of ten different sandstone and carbonate samples (5 mm in diameter and 10 mm in length) at four different voxel resolutions. Porosity and permeability of all samples were calculated using images analysis and predicted by pore-network and lattice Boltzmann modelling, respectively. Their results showed that the pores and throats smaller than the scanning resolution are blurred in the images and could be wrongly assigned to intermediate phase or grain phase which affects the accuracy of calculated petrophysical properties.

Advantages and limitations of X-ray CT are summarized in Table 2. The main advantages of this technique include being a non-destructive technique, allowing for 4D (temporal and spatial) monitoring of internal structures and having high resolutions down to a few hundred nanometres. Operator dependent analysis, the discretization effects, and imaging artifacts have been reported as limitations of X-ray CT [14]. 

Singh et al. [196] studied the dynamics of displacement events, such as snap-off and pore- filling, during imbibition at reservoir pressure conditions. They also compared local capillary pressure variations during a snap-off event in drainage and imbibition processes. High-resolution (voxel size of 3.28 µm) and fast (38 s between each image) synchrotron X-ray micro-tomography was used to visualize the swelling of the brine layer leading to a snap-off process. They performed a drainage (oil displacing brine) followed by an imbibition test using a 3.8 mm diameter and 10 mm long Ketton limestone sample. During both experiments, continuous high-resolution imaging was performed to capture displacement events. Their results showed that snap-off events in imbibition with time scales of several minutes are significantly slower than Haines jumps in drainage. 

Contact angle measurements on flat surfaces of minerals at ambient conditions have been used as a classical method to define the wetting state of rock surfaces. With the recent improvements in X-ray µCT imaging and processing, it is now feasible to perform in-situ contact angle measurement or direct test of surface wettability at pore-scale [215,223,224,225,226,227,228].

#### 3.2.2. Neutron Tomography

Neutron tomography has similar basic principles to that of X-ray tomography, with the exception that a beam of neutrons is used instead of X-rays [229,230]. Similar to X-ray tomography, neutron imaging is a non-destructive technique that allows internal structures to be visualised in 3D. Multiple two-dimensional images are taken and 3D images are then constructed using mathematical algorithms and computers [231]. 

Neutrons interact with the atoms’ nuclei, whilst X-rays interact with the electrons of the atomic shells. Materials containing metals have higher X-ray attenuation, whereas neutron attenuation is higher in materials containing hydrogen, which makes neutron tomography applicable for identification of light materials inside the sample of interest [232,233,234]. 

Improvements in resolution and quantum efficiency of imaging detectors have helped to provide spatial resolutions of less than 20 µm with field of view of 33 mm, as presented in Table 2 [235,236]. However, acquisition time is significantly affected by the resolution and there is a compromise between good resolution and acquisition time. With pixel resolution of 20 µm and a detection efficiency of 90%, a single tomography image can take up to 25 h [236]. Recently, Kaestner et al. [237] developed a set of testing devices to measure neutron image resolution, pixel size and beam divergence. Using their simple and efficient method, the resolution of a neutron imaging set up can be described, although it might be complicated to develop similar devices for high resolution (in the order of a micron) systems.

Vontobel et al. [232] compared X-ray tomography and thermal neutron tomography methods used to study geological materials; a small ammonite and one diamond bearing eclogite (a metamorphic rock type). They concluded that these two methods give complementary information, as neutrons can visualize materials containing hydrogen and components with low atomic number that cannot be seen by X-rays.

Although neutron imaging produces images with less resolution than X-rays, it has a high penetration depth and can be used for samples with the size of one to a depth of several centimetres, which allows studying large sample. Using neutron imaging it is possible to image a sample of 100 cm^3^ volume at a medium resolution (down to 30 µm) which is not possible by using X-ray [238,239]. Neutron tomography can also provide more information about a sample structure compared to X-ray tomography, when the sample consists of components with the same X-ray attenuation, such as iron or titanium [234]. 

Neutrons are sensitive mainly to light elements such as hydrogen, as a results the neutron imaging can detect water distribution in porous media, and therefore, volumetric and temporal phase distributions can be monitored [229,230]. Neutron imaging is ideal to detect small amounts of water down to droplet sizes of 30 μm or water layers as thin as 10 μm [231]. Potential applications of neutron imaging in geosciences for quantifying textures of deformed crystalline rocks, studying multi-phase flow, CO_2_ sequestration, and investigating the distribution of organic and inorganic carbon in silicate and carbonate rocks were presented by Wilding et al. [233]. 

Generally, the neutrons are slowed down by passing through a moderator which either consists of cells of water at room temperature or containers of hydrogen to produce a thermal or cold neutron beam, respectively. The thermal neutrons have a much higher energy than the cold neutrons that results in very small absorption cross-sections and consequently larger samples can be scanned. This makes the thermal neutrons more interesting for scanning geological samples [229]. The application of neutron tomography in geosciences is gaining increasing interest, as neutrons have more depth of penetration than X-ray and can track flow front, and moreover, there has been significant improvements in reducing acquisition time and higher resolution.

#### 3.2.3. Positron Emission Tomography (PET)

Positron emission tomography (PET) is a medical imaging technique categorized under nuclear medicine imaging. PET uses small amounts of radioactive material called radiotracers or radioactive tracers, which propagate through the sample body and emit positrons. The emitted positrons annihilate with electrons in the sample and as a result two photons are emitted. A detector is required to detect pairs of photons and the spatial distribution of a radiotracer is obtained by computer tomographic reconstruction [240]. With the exception of the injection of the radiotracer, PET is a non-invasive and non-destructive imaging method. Brownell and Sweet [241] developed the first PET prototype in 1953, but the main progress happened in the 1970’s.

The spatial resolution of clinical PET-scanners is usually around 3–5 mm as presented in Table 2 [242,243]. Some biomedical PET-scanners are designed with a smaller FOV achieving a resolution of around 1 mm [244]. Khalili et al. [245] and Boutchko et al. [242] investigated application of PET for studying fluid flow in porous media (sandy sediments, packings of spherical glass beads and sand packs). They successfully used PET to visualize 3D flows inside natural and artificial porous media. PET has been combined with CT leading to more precise and detailed information on the fluid transport processes [246,247]. They found PET-CT imaging very applicable to visualize spatial and temporal distribution of fluid and front progress.

Parker [248] recently presented radioactive particle tracking (RPT) and positron emission particle tracking (PEPT). PEPT is a technique for three-dimensional (3D) tracking of single radioactively labelled particles moving at high speed (metres per second) inside a dense object.

PET has gained interest in different research areas of reactive transport and geochemistry in geosciences and subsurface energy engineering, especially in combination with X-ray CT. High temporal resolution and signal-to-noise ratio as well as negligible effect of radiotracer on the flow has made PET a unique technique to quantify solute mixing and spreading [248]. The temporal resolution of 10 s and high sensitivity of PET systems to the presence of a radiotracer make it possible to monitor processes occurring at small features in porous media, including fractures. Zahasky et al. [243] reviewed applications of PET for research in water and subsurface energy resources. The focus has been on improving the resolution of PET detectors, exploiting new mechanisms for prompt photon emission, and developing improved photodetectors [240]. 

#### 3.2.4. Nuclear Magnetic Resonance Imaging (NMRI) or Magnetic Resonance Imaging (MRI)

The principles of nuclear magnetic resonance (NMR) were established in 1946, and its use for constructing images was introduced in 1971 [249,250]. Two years later, Lauterbur [251] proposed a method of producing 2D images using NMR technique. The first medical image of a finger was produced by Mansfield et al. [252] in 1974. Lauterbur and Mansfield received a Nobel Prize in 2003 for discovering of NMR based multi-dimensional imaging technique [253]. 

The spinning of certain charged nuclei yields a magnetic moment and once an external magnetic field is applied, the nuclear magnets are oriented in the direction of that field. By applying a low energy pulse with a proper frequency, the sample absorbs some of the energy, and as a result, the sample’s magnetic moment deviates from the magnetic field. The magnetic moment starts a motion which causes the emission of energy in the form of a radio signal with the same frequency as the applied energy pulse. Five variables of spin density, T1 (longitudinal) and T2 (transverse) relaxation times, flow, and spectral shifts are measured and used to construct the images [250,254]. 

Nuclear Magnetic Resonance Imaging (NMRI) or simply magnetic resonance imaging (MRI) is a non-invasive and non-destructive technique and provides images of high spatial resolution comparable to X-ray tomography, but different information is contained in the obtained images. Strong magnetic fields, radio waves, and field gradients are used in MRI and the acquisition time is usually longer than CT [255]. The image resolution and time required to acquire MRI images depend on size and characteristics of the studied sample and scanner specifications. As presented in Table 2, the resolution of MRI images can go down to a few tens of μm for sample size in cm-scale (core scale) but it decreases to mm’s for m-scale samples [5,193]. 

NMRI advantages have made this technique interesting to different applications. No ionizing radiation is employed in NMRI and instead radio waves and magnetic fields are used, and therefore, the associated risk of radiation is eliminated. Only fluids are visible in NMRI and it provides excellent tissue contrast using differences in the density and the molecular environment and as a result, there is no need for injecting toxic contrast agents. Steinberg and Cohn [256] summarized the main limitations of NMRI techniques compared to X-ray as high expense installation and imaging and more acquisition time, which are presented in Table 2. 

NMRI has been applied for formation evaluation and core analysis purposes, especially at reservoir conditions [257]. NMRI has been used to characterize porous media properties (pore size distribution, porosity, and grain size), to determine fluid distribution in porous media, to measure solute and fluid transport properties (flow paths and velocities), and to study reactive transport (reaction kinetics and reactant distribution) [5]. 

In summary, NMRI has been applied in a wide range of studies in transport in porous media and geomaterials. This technique can be used for direct 1D, 2D, and 3D imaging of processes and it is possible to distinguish different chemical species in the system. The spatial and temporal resolutions of this technique depend on the strength of the magnetic field and gradient strength. Unfortunately, a magnetic resonance scanner can be very expensive with high maintenance cost.

#### 3.2.5. Gamma Radiation

Gamma rays are electromagnetic high-energy photons, which originally were discovered by Becquerel in 1896. Gamma rays travel at the speed of light and have a shorter wavelength (<10 pm) than electromagnetic radiation emitted by X-ray tubes. The main difference between gamma rays and X-rays is their origin, where gamma rays are emitted by the nucleus, whilst electrons orbiting the nucleus emit X-rays [258]. Single or dual energy gamma rays, as a non-invasive and non-destructive method, can be used for studying the bulk density and wettability of rock and soil samples [259,260,261,262]. Single energy gamma rays was used by Ursin [263] to obtain the local water saturation over a cross-sectional area in two-phase flow experiments performed on a heterogeneous porous medium of unconsolidated glass powder. In the dual-energy gamma radiation, 241-Americium and 137-Cesium are usually used as low- and high-energy gamma ray sources, respectively [264]. The gamma beams from the two energy sources are simultaneously emitted and travel through the same sample section. They pass a common detector collimator and then reach the detector. Two separate images are generated, and material composition can be determined by calculating a relative ratio of energy absorbed. Werth et al. [5] described different components of a dual-energy gamma system including source holder, detector, and vertical movement configuration were demonstrated. Dual-energy gamma radiation has been used to study Darcy scale processes in porous media with varying size of centimetres to meters [265,266]. 

### 3.3. Electron Microscopy Methods

Electron microscopy imaging techniques have been employed to improve visualization of a broad range of biological, environmental and geological processes at very small scales down to nanometres. In this section, two commonly used microscopic techniques in geosciences are reviewed, namely transmission electron microscopy (TEM) and focused ion beams scanning electron microscopy (FIB-SEM). Table 2 summarizes features of these methods and compares them with other imaging techniques discussed in this review.

#### 3.3.1. Transmission Electron Microscopy (TEM)

TEM is a microscopy imaging technique in which a beam of electrons is transmitted through an ultrathin section of an object, usually less than 100 nm. Knoll and Ruska constructed the first electron microscope prototype in 1931 and won the Nobel Prize in 1986 [267]. A TEM has an electron emission (illumination) source, electromagnetic lenses and a projection system. The electron beam is produced, accelerated and focused on the sample by the lenses. The beam passes through the sample, which modifies it and imprints its image. The projection system enlarges the image and projects it onto a viewing screen (electron detector) such as a fluorescence screen. An imaging device is used to magnify the taken image and focus on the area of interest [268]. Another type of electron microscopy is scanning transmission microscopy (STEM) in which the electron beam is focused on a specific part of the sample and scans the whole surface stepwise. STEM is used only when the information about the sample surface is required. It must be noted that the sample in STEM should be larger or thicker than TEM [269].

The wavelength of moving electrons is several orders of magnitude smaller than the wavelength of visible light and therefore, the imaging resolution of TEM is significantly higher than light microscopes. TEM can capture a single column of atoms, which makes this technique a major analytical method in biological, chemical and materials sciences for different applications such as research in cancer, nanotechnology, pollution, and semiconductor [270]. Wu and Aguilera [271] reported instrument cost, limited access, image processing time and quantitative application as the challenges of using this technique. Although the operating scale of TEM is not in a range of a representative elementary volume (REV), which is suitable for fluid flow studies in porous media, valuable information about pore structure and connectivity can be obtained at very low scale, helping to understand the flow mechanisms [12]. 

#### 3.3.2. Focused Ion Beams Scanning Electron Microscopy (FIB-SEM)

Focused ion beams scanning electron microscopy (FIB-SEM) integrates a focused ion beam (FIB) with a scanning electron microscope (SEM) to acquire detail information on the internal structure of solid objects [272]. 

Cambridge Scientific Instrument Company (Cambridge, UK) built the first commercial SEM instrument in 1965 [273]. SEM is a powerful technique for 2D imaging with high resolution between 1 and 20 nm. However, it does not provide any image in the third dimension, which can be a limitation for application of this technique. Therefore, SEM is sufficient to image the pore space, but it cannot be an appropriate technique for studying pore volume and connectivity [4,274]. As mentioned in the previous section, Curtis et al. [275] used SEM techniques in combination of other techniques to understand pore structure and connectivity of shale samples. SEM and TEM were employed to investigate shale gas at nanoscale and propose a petrophysical model for calculating water saturation in shales [271].

FIB was developed in 1975 and its initial applications were etching, ablation and deposition of material on solids, but it rapidly became a popular technique in the semiconductor industry [276]. FIB operates similarly to SEM, except that a focused beam of gallium ion is used instead of a beam of electrons. Moreover, unlike SEM, FIB is a three-dimensional (3D) imaging technique with resolution down to 1 nm. Although FIB provides 3D high resolution images, it exposes only small areas of observation (e.g., 20 µm at high magnification) and cannot provide adequate information for characterizing a sample especially for heterogeneous samples [272]. 

Combining FIB and SEM provides multi-scale imaging capabilities and in particular, for porous media it allows for visualization of meso pores (2–50 nm) and macro pores (>50 nm) [277]. FIB-SEM acquires high resolution 3D images and can achieve voxel dimensions of 50 nm or less but only for very small samples in order of few µm [4,275,278]. 

Considering that the feature sizes in porous geomaterials are in a wide range, from nanometers to centimeters, De Boever et al. [279] suggested a workflow for a chemical and structural characterization of a representative volume of heterogeneous geomaterials. In proposed workflow, information obtained from X-ray CT at different spatial resolutions are combined with information derived from scanning electron microscopy and energy-dispersive X-ray spectroscopy.

The scale of representative REV for this technique is not appropriate for visualizing flow in porous media, but vital information can be obtained about pore structure and connectivity which helps to understand fluid flow mechanisms and build pore-scale numerical models for upscaling purposes [12,274,279].

## 4. Applications of Micromodels and Imaging Techniques

This section discusses the four main applications of micromodels and imaging techniques relevant to geoscience, hydrogeology and petroleum engineering, including fluid distribution and displacement (Section 4.1), fluid flow in heterogeneous and fracture media (Section 4.2), reactive, solute, and colloids transport (Section 4.3), and porous media characterization and deformation (Section 4.4). Special focus is placed on fluid distribution and displacement, as this is particularly relevant for fluid flow in porous media. Table 3 summarizes different applications of both micromodels and imaging technique in geoscience and geo-energy engineering with the most relevant references which have been reported in this paper.

### 4.1. Fluid Flow in Porous Media (Drainage, Imbibition, Front Evolution, Phase Trapping)

Immiscible fluid displacement in porous media happens in many processes, such as surface water infiltration into soil (water displacing air), underground water contamination (pollutants displacing water), enhanced oil recovery, EOR (water or gas displacing oil), and CO_2_ storage (e.g., CO_2_ displacing water or oil). Three main forces, namely viscous, gravity and capillary control the displacement processes. Darcy’s law, which was first introduced by Henri Darcy in 1856 [280], is the equation that governs the fluid flow in porous media and basically demonstrates that the total flow rate is proportional to the total pressure drop across the media. Darcy’s law was extended for a two-phase flow by introducing relative permeability of each phase to the original equation as shown in Equation (2).

(2) $$q_{i}=-\frac{kkr_{i}}{\mu_{i}}A\left(\nabla P_{i}-\rho_{i}g\right)$$

where *k* and A are the permeability and cross-sectional area of porous media, *q_i_*, *kr_i_*, *µ_i_* and ρ*_i_* are the flow rate, relative permeability, viscosity and density of phase *i* respectively, ∇*P_i_* is the pressure gradient in phase *i* and g is the gravitational acceleration. 

The effect of capillary forces on multiphase flow at Darcy-scale is captured by the capillary pressure (*Pc*). *Pc* is the difference in pressure across the interface between two immiscible fluid phases and is mathematically defined as a function of phase saturations. Although both *kr* and *Pc* are Darcy-scale flow functions which are used as input parameters to the numerical simulation models, they are influenced by micro-scale parameters such as pore size distribution, pore surface wetting states. Pore-scale imaging and modeling have been used to predict *kr* and *Pc* curves. This technique is known as digital rock physics (DRP) [281,282,283]. It is known that the multi-phase flow in porous media is a function of viscosity ratio of immiscible fluids, density, interfacial tension, heterogeneity, wettability and pore surface roughness [10]. Therefore, understanding the effect of each parameter on fluid flow and displacement at the micro-scale is essential to understand, model. and predict the flow functions (*kr* and *Pc*) and subsurface processes at larger scales.

In this review, we analyse flow experiments using micromodels and in-situ imaging to understand flow mechanisms and validate mathematical models. The direct observation of the events at pores and channels has been an integral part of our fundamental understanding of the governing mechanisms at both micro- and macro-scales. Pore-scale visualization techniques have contributed significantly to identify mechanisms such as capillary and viscous fingering, pore/capillary filling, snap off trapping, bypass trapping, and film flow. Most of the published work uses one technique and only a few studies integrate micromodel testing with 3D imaging techniques. 

#### 4.1.1. Effect of Pore Network Pattern

Various flow network patterns similar to some of the patterns shown in Figure 1 (including regular and irregular triangular patterns) in PDMS models were used to study the effect of the interfacial area on the displacement efficiency of fluids [32]. The aim of these experiments was to simulate the drainage process of oil from a bypassed oil-wet zone during water flooding in a heterogeneous formation. The regular flow networks were constructed from either hexagons, squares, diamonds or triangles, whereas the irregular flow patterns were generated using an algorithm based on two-dimensional Voronoi diagrams. The depth of channels was constant (around 15 μm) in all models, whilst the channel width was constant (either 6 μm or 8 μm) in the models containing regular patterns or was varied (in the range of 4–8 μm) in the models containing irregular patterns. All these models were characterized by different coordination numbers, i.e., the number of channels connected to every interior pore body, which were found to have an impact on water-oil displacement efficiency. 

Tsakiroglou and Avraam [48] constructed models with the use of an excimer laser and the LIGA process. The depth and width of pores and throats (channels) were obtained with accuracy better than 5 μm and 10 μm, respectively. Since the channels were generated with a laser beam, the intersection regions (nodes) were as deep as the sum of the two intersecting channels. The authors expected that the capillary properties of these models will be similar to the capillary properties of naturally porous formations, such as sedimentary rocks and soils. The models were used in simple imbibition experiments in which n-decane (wetting fluid) was injected into the pore space in order to displace air (non-wetting fluid). Chapman et al. [284], in turn, investigated both spontaneous imbibition and drainage at the level of pores with the use of PMMA micromodels. For this purpose, specifically designed micromodels were used to study the impact of pore shape and throat width on fluid displacement. To achieve different capillary entry pressures (from 1.17 to 2.2 kPa, which is equivalent to 11.5 to 21.9 mbar), the micromodels comprised channels of different widths. During the drainage experiments, it was observed that the fluid displacement in the junctions follows the Young-Laplace law, whilst the imbibition experiments using the micromodels comprising unequal channel widths showed that fluid displacement does not follow capillary filling rules. Instead, the filling sequence was found to be dependent on the pore geometry, and specifically channel proximity, suggesting that current network models for spontaneous imbibition may not accurately predict fluid displacement pathways.

Karadimitriou et al. [27] constructed models in which the pore network topology was generated using Delaunay triangulation, which was considered to provide a good representation of real porous media. The models comprised pores with a size of approximately 40 μm, which were connected by channels (pore throats) of different widths. In their drainage and imbibition experiments, the capillary pressures never exceeded 6.2 kPa (0.06 bar) to avoid deformations of micro-channels within the micromodel structure. The conducted experiments concluded that for two-phase or multi-phase flow, the interfacial area should be included as one of the state variables, in addition to pressure and saturation to eliminate hysteresis for drainage and imbibition They could estimate a unique value of interfacial area for every *Pc*-saturation pair. The interfacial area is the total area of contact between two fluid phases in a two-phase flow system. 

Li et al. [285] studied two-phase flow of liquid CO_2_ displacing water (drainage) in a silicon-glass micromodel with heterogeneous pattern and in order to capture fluid displacement patterns and abrupt changes in the velocity field, µPIV and fluorescence microscopy were employed together. The visualization methodology was originally proposed by Kazemifar et al. [286]. They seeded water with fluorescent particles and tagged liquid CO_2_ with a fluorescent dye. As a result, they could instantaneously measure the temporal and spatial velocity field of water and capture spatial configuration of both phases during the displacement. They could also capture the propagation of fingers and events like Haines jumps and calculate the corresponding local Reynolds number. 

A silica-based micromodel representing a replica of the Berea sandstone pore pattern was used by Buchgraber et al. [121] for the investigation of gas trapping mechanisms occurring during the imbibition of water into a CO_2_-saturated system. The micromodel enabled experiments at two different temperatures (295.35 K and 317.55 K) and four different pressures between 0.076 and 7.93 MPa (0.76 and 79.3 bar). Using this micromodel, it was possible to observe trapping mechanisms for three different phases of CO_2_ (i.e., gas, liquid and supercritical) at the pore level. Keller et al [119] generated a two-dimensional replica of the Berea sandstone cross-section on the surface of a silicon wafer and used this micromodel to observe displacement mechanisms at the pore scale for three-phase flow (water-oil-air). The model contained an irregular network of pores, which were around 15 μm deep and 3–30 μm across. A similar micromodel containing a replica of the Berea sandstone network pattern was also exploited for the investigation of two-phase flow mechanisms for two fluid pairs (air-water and decane-water) and for the observation of the behaviour of wetting and non-wetting fluids flowing through irregular pores [18]. 

#### 4.1.2. Front Instability

In the 1950s, the glass-bead models were mainly exploited for the investigation of macroscopic instabilities (so-called “fingers”) generated by two immiscible fluids (oil and water) in regular and quasi-regular porous networks. These models provided a useful insight into viscously driven instabilities and enabled the validation of an existed instability theory [64,73,287]. Since then, the glass-bead models have found many new applications in geological and geo-energy engineering research. For instance, Wang [75] conducted experiments with a high-pressure glass-bead-packed flow tube to observe the physical phenomena of the displacement of crude oil by CO_2_ under miscible, semi-miscible and immiscible conditions. Lu et al. [288,289,290] published a series of papers in which glass-bead models were used to study the movement of water in a soil system, thereby enabling the understanding of many flow-related processes, such as the capillary rise, fingering flow, and infiltration. Glass-bead micromodels have been used to investigate different techniques for improving the efficiency of water flooding in porous media [291]. High-pressure experiments using a silica-based model were also performed by Wang et al. [123] to investigate the mechanisms that affect the displacement of supercritical CO_2_ by water under reservoir conditions. These experiments were conducted at a temperature of 314.15 K and under a pressure of 9 MPa (90 bar). The micromodel comprised a regular network of 200 μm diameter cylinders, spaced approximately 230 μm apart. The same (or at least very similar) micromodel was used for the verification of simulation results involving the viscous fingering, capillary fingering, and stable displacement of immiscible fluids in porous media [124]. Oxaal [292] used a photoresist-based micromodel to study fingering effects caused by fluids of two different viscosities in heterogeneous porous structures. The common feature of all these experiments was that they were conducted with low injection pressures, i.e., <100 kPa (1 bar) above the ambient conditions. This was necessary to avoid damage of the photoresist. Although more irregular porous media can be studied using photoresist-based micromodels, the pressure range is more limited, and it is strongly recommended to consider this limitation during the experimental design.

Haugan [293] constructed a two-dimensional PET system to image viscous fingering during immiscible displacement in porous media. A Clashach sandstone core with a diameter of 3.81 cm and length of 7 cm was used. They injected sea water at a rate of 6 cm^3^/h into the vertically oriented core to displace oil. A tracer was injected in front of water to be the interface between displacing (water) and displaced (oil) phases. They clearly visualized the fingering effect during the displacement and proved PET can be a powerful imaging tool in geosciences and engineering applications.

#### 4.1.3. Saturation Distribution & Trapping Mechanisms

Glass micromodels were used to investigate the displacement of oil by CO_2_ at ambient temperature, both at the presence and the absence of water, under low- and high-pressure conditions [99,110]. High pressure experiments were conducted by placing the micromodels in a pressurised vessel filled with glycerine. Although the pressure of the injected CO_2_ was very high (8.27 MPa which is equivalent to 82.7 bar), the difference between the pressure inside the micromodels and outside (but inside the vessel) was small. A pressurised vessel was also used to conduct experiments with glass micromodels under really high-pressure conditions up to 35.16 MPa or 351.6 bar [110]. These micromodels were used to investigate the residual oil recovery mechanisms when different fluids (e.g., near-miscible gas or low-salinity water) were injected into the porous system. Riazi et al. [117] used glass micromodels to study of mechanisms involved in CO_2_ injection and storage in hydrocarbon reservoirs and water-bearing aquifers at pressures up to 13.79 MPa (137.9 bar). The effects of wettability changes induced by crude oil on the distribution of residual oil in porous media were also studied by using glass micromodels [98,102]. More recently, it has been demonstrated that pore network models made of fused silica can be used to observe changes in wettability of minerals and rocks upon their reactions with supercritical CO_2_ and brine [103], as well as to investigate the wettability effects on the displacement of brine by supercritical CO_2_ during drainage [104]. In both cases, the models contained a homogenous two-dimensional pore network pattern which was composed of 590 μm diameter cylindrical pillars spaced apart by 640 μm. The experiments were carried out at a temperature of 318.15 K and pressure of 8.5 MPa (85 bar). 

**Figure 7:**
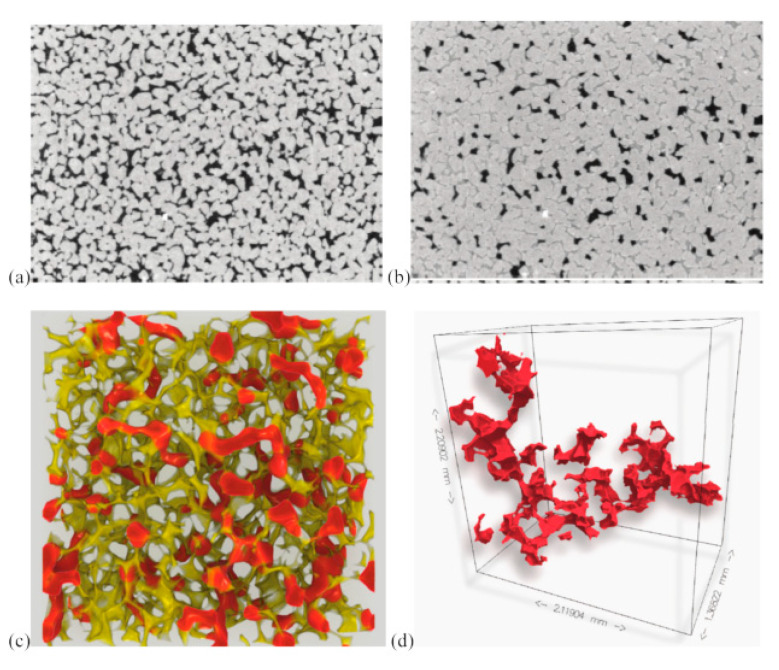
µCT images (2D and 3D) for a spontaneous imbibition (SI) in a strongly water-wet saturated with gas. (**a**) cross-section of a dry Fontainebleau sample, (**b**) the same cross-section after SI, (**c**) 3D visualization of a small sub-section of the 3D volume at residual gas saturation; the yellow shows the pore structure and red the trapped gas phase. (**d**) a 3D rendered image of a single large gas residual blob. (Reprinted with permission from Kumar et al. [294]).

Tomography techniques have been used extensively to monitor and visualize fluid flow phenomena and physics of flow at pore-scale. Kumar et al. [294] studied effects of initial water saturation, flooding rate and rock wettability on the hydrocarbon trapped phase in the imbibition processes using X-ray µCT. They performed series of imbibition experiments on sandstone and carbonate rock samples with 5mm diameter and at least 2 cm length. Thereby, 3D image volume of 20,483 voxels were reconstructed and the residual saturation of non-wetting phase in the network of pores was measured using the images. Figure 7a,b shows a slice of a strongly water-wet sandstone core sample before and after a spontaneous imbibition experiment, respectively. Figure 7c,d shows residual gas saturation (in red colour) in a small 3D subsection of the core after the experiment. They observed that the non-wetting phase was trapped in the larger pores and the wetting phase was in the smaller throats. Information about microscopic distribution of hydrocarbon trapped phase in a bearing formation can help to understand multiphase flow and displacement mechanisms. Local oil saturation and size distribution of oil ganglia have been measured by Oughanem et al. [295,296] using X-ray µCT to study the effect of pore geometry, interfacial tension, and flooding parameters on the performance of surfactant injection. 

Imaging fluids distribution in porous media is one of the main applications of gamma radiation imaging method. Oostrom et al. [297] used a dual-energy gamma radiation system to determine non-aqueous phase saturation distribution in a fine-grained sand pack. They compared the obtained saturation distribution with results of numerical simulations and concluded there is a shortcoming with current relative permeability-saturation-capillary pressure models in established multiphase flow simulators. Brusseau et al. [298] investigated mass flux reduction, mass removal and their relationship during remediation processes, where immiscible liquids as source of subsurface contamination are poorly accessible to flushing water. They designed rectangular and cylindrical flow cells with 50 cm and 10 cm lengths, respectively, and used natural sand as the porous media. A dual-energy gamma radiation was used to measure saturation of immiscible liquid saturations in the flow cells and map the distribution. They reported spatial resolution of 0.25 cm^2^ and sensitivity of 0.003 for an average saturation measurement over the width of the flow cell.

#### 4.1.4. In-Situ Quantitative Measurements 

Recently, Oostrom et al. [125] constructed a set of silicon-glass micromodels that comprised partially-regular pore network patterns. The observation of processes occurring inside the pores was performed by using an epifluorescent microscope (a type of fluorescence imaging) and a ×10 inverted objective. This optical arrangement enabled the capture of images with a 0.65 μm resolution and provided a sufficient contrast between two different fluids. They conducted four different sets of non-reactive solute transport experiments, in which only one parameter (flow velocity, grain diameter, pore-aspect ratio or flow-focusing heterogeneity) was used as a variable. The experimental data sets were then offered to various pore-scale modelling groups to train and test their numerical “pore-scale” simulators. By comparing the simulation results with the experimental data, it was possible to verify different pore-scale numerical models. Although the qualitative analysis of captured and visualized fluid flow events at the pore-scale has substantially improved the modelling of transport phenomena in porous media, in-situ quantitative measurements are still essential to our mathematical modelling at both pore and continuum scales more accurate. Micromodel testing and imaging techniques have been employed to measure the spatial and temporal variations of velocity, pressure, shear stress, phase saturations, contact angles, mineral dissolution and precipitation at pore-scale to compare them with simulation results. 

Roman et al. [171] performed µPIV measurements in silicon-based micromodels with regular and complex pore patterns. They used a microscope and several objective lenses with different magnification and numerical aperture to be able to track the motion of the fluids at different scales. A Metal Halide lamp as the light source and a CCD were used to acquire images. They presented the pore-scale velocity distributions for a single-phase flow at pore size of 5–40 μm and performed comparisons between experimental and simulation results. Moreover, the dynamic of immiscible two-phase flow was studied using µPIV measurements. Heshmati and Piri [179] developed a new two-phase and two-fields-of-view μPIV experimental apparatus to investigate velocity fields, shear stress at the fluid/fluid interfaces and the trapping and reconnection mechanisms at two-phase flow conditions in PDMS micromodels. Study of fluid movement in certain pores and across the entire micromodel was possible due to integration of small and large FOV modules with the apparatus. Most recently and for the first time, Roman et al. [173] investigated the magnitude of the interfacial momentum transfer force for different flow conditions using a μPIV experimental set-up. 

Al-Mugheiry et al. [299] used fast NMRI (typically 0.1 s per image) to image fluid flow in sandstone core samples, sand and glass-bead packs. They showed that this high-speed snap-shot technique have a great potential to be used for quantitative measurements of spatial flow in porous media. Fluid flow and dispersion in porous media were studied by Manz et al. [300,301] using NMR velocimetry measurements and lattice-Boltzmann modelling. They used three unconsolidated packings of glass beads of different diameter as the porous media and injected I-mM aqueous CuSO_4_ solution at constant rates in the range of 4–100 cm^3^/h. They obtained 2D velocity maps from NMRI that were in good agreement with simulation results obtained from lattice-Boltzmann. Bijeljic et al. [302] used MRI velocimetry to investigate macroscopic and local velocity field in creeping flow of a Newtonian fluid in a fibrous porous media. They obtained steady-state velocity maps of the longitudinal and transverse velocity components of the flow field in such a heterogeneous medium. Nicholls and Heaviside [303] employed the gamma-ray-absorption technique to measure in-situ fluid saturation and found it superior to volumetric and gravimetric material balance methods. They showed that measuring in-situ pressure and saturation profiles improves the analysis of core displacement tests, such as relative permeability measurements. Fluid front evolution and saturation distribution in oil displacement processes were visualized by Huang and Gryte [304] using gamma radiation for thin slabs of porous media.

Recently, Zarikos et al. [305] manufactured a PDMS micromodel with integrated fibre optic pressure sensors to measure pressure at the pore scale. They used soft lithography for manufacturing the PDMS micromodels. Miniature fibre optic piezometers (FOP-MIV) which are usually used for fluid pressure measurements in live tissues were embedded in the micromodel. These sensors have a diameter of 260 µm and were protected with a cover sleeve which increased their OD to 320 µm. The measurement range of the used sensors was from −40 to 40 kPa with a resolution of 4 Pa and accuracy of 0.6% of the full range. They monitored the pore pressure change of pore-filling events and also the breakthrough time when the fluids reach the micromodel outlet. Pore-scale pressure measurements can be used to calibrate and improve pore-scale numerical models, leading to more accurate predictions of multi-phase flow in porous geomaterials. 

A small change in thickness of a PDMS substrate can imply a substantial change of the light intensity that passes through the layer. Turek et al. [306] investigated the effect of deformation on the optical properties of the PDMS and showed that the effect of compression on the optical transparency and refractive index of PDMS is significant. They concluded that PDMS could be employed for constructing stress optical sensors of mechanical displacement or stress. Moreover, Hosokawa et al. [307] used the PDMS deformable diffraction grating to monitor local pressure in a microfluidic device. They monitored the pressure by detecting the change in optical properties of the grating. Their test device containing a diffraction grating and a microchannel could produce sufficient optical response to air pressure ranging from −80 to 100 kPa.

Singh et al. [139] used confocal Raman spectroscopy to conduct in-situ mineral characterization of a 500 µm-thick section of a real rock sample before and after embedding in a PDMS microchannel. Poonoosamy et al. [153] integrated a microfluidic chip with high-resolution imaging including optical microscopy and Raman spectroscopy for in-situ, non-destructive and real-time monitoring of chemical and transport processes. X-ray µCT imaging has recently made it feasible to perform in-situ contact angle measurement feasible [215,223,224,225,226,227,228]. Factors such as grain roughness and mineral heterogeneity within the pores can affect contact angle values at the pore-scale [308,309,310,311]. Moreover, capillary dominated flow processes are strongly influenced by the wetting state of pore surfaces. Therefore, a realistic distribution of contact angles is profoundly important to define local wettability characteristics of porous media in pore-scale computational models [312].

### 4.2. Flow in Heterogeneous Rocks and Fractures

An in-depth understanding of fluid flow, mixing, and reactive transport is key in heterogeneous porous media and fractures for a wide range of applications. Spatial heterogeneity in pore and continuum scale medium has a significant effect on local and large-scale processes. Both techniques of micromodel experimentation and in-situ 3D imaging have been used to characterize heterogeneous rocks and fractures for a better understanding of transport phenomena in these media.

#### 4.2.1. Fractures Characterization

Glass micromodels have been used to study fluid flow in fractured porous media [108,109,111]. For instance, Kamari et al. [109] constructed glass micromodels containing different fracture geometries within a pore network pattern and used these models to investigate the effect of the fracture length and its orientation on the breakthrough time during the miscible displacement process using n-heptane and n-decane. The effect of fracture geometry on the oil recovery efficiency during the injection of miscible fluids (such as n-hexane, n-decane, and mixed solvents) was also studied by Farzaneh et al. [111]. 

Tomography has been a very useful tool for the investigation and characterization of fracture networks in porous media. Determining fracture aperture and understanding flow pattern in a network of pores and fracture is of great interest. Van Geet and Swennen [197] visualized fracture patterns in 3D and measure fracture aperture at any location using X-ray µCT for samples with 8 mm diameter. A standard deviation of 15 µm was reported for measurement of fracture apertures of 100 µm in coal samples and it was found that the scattering due to high attenuating particles can cause anomalies in the measurements. High resolution industrial X-ray scanners was used by Liu et al. [198] to study the cracking process in Longmaxi formation shale. They examined the evolution of the fracture network and the failure micromechanics in the layered shale as a function of the inclination angle of the bedding plane [198]. Lewis et al. [313] performed flow tests on low permeability carbonated rock samples (laminites), which were experimentally fractured, while using neutron radiography and tomography for imaging flow pattern. They observed that the injected fluid moves up and down in the fracture network and invades the matrix where connected to the network. As expected, the front progression in fractures was slow for less developed network areas. Dijk and Berkowitz [314] used NMRI to measure flow patterns in naturally fractured rocks and study effect of fracture morphology on flow pattern and evaluate existing models. They induced artificial rough fractures, with the mean aperture of around 2 mm, in limestone samples, performed horizontal water flooding through the samples and obtained 3D velocity vectors. They found wall roughness and sharp fracture wall discontinuity as effect parameters on velocity profiles and flow pattern complexity.

The application of PET for visualization in fractured and heterogeneous media has recently gained more attention as a good and efficient method. PET can detect highly penetrating radiation which helps to visualize flow at high pressure and high temperature or reservoir conditions, where pressure vessels with very thick metal walls are used. PET can be used in conjunction with other conventional techniques providing very useful supplementary information [293]. Kulenkampff et al. [315] studied anisotropy and heterogeneity in clays using PET. They used a core of 10 cm diameter and 8 cm length which is a good representative size to capture structural features. They recorded the spatio-temporal evolution of the tracer distribution and derived anisotropic diffusion coefficients. Heterogeneity evaluation was performed based on changes in the tracer concentration in which a zone with higher concentration identified as more heterogeneous. Later, they reviewed and discussed applications of PET in geoscientific studies and presented examples of monitoring advection and diffusion processes [316]. They introduced their upgraded PET scanner having higher resolution (1 mm) and sensitivity than the medical scanners and with a larger FOV (maximum diameter and length: 160 mm and 110 mm). They found that the image quality of clinical PET with a resolution of 3–5 mm is poor for a core sample diameter of 10 cm. 

#### 4.2.2. Drainage & Imbibition in Fractures 

Rangel-German and Kovscek [18] used a silica-glass micromodel containing a replica of the Berea sandstone network pattern with fractures to investigate the behaviour of wetting and non-wetting fluids flowing through irregular pores and fractures. Recently, experimental studies were conducted with PMMA micromodels to investigate the non-Darcy interfacial dynamics of two-phase flow (water and air) in rough fractures under drainage conditions [317,318]. The fractures with an average width of 2 mm and depth of 4 mm were generated on PMMA plates using a CO_2_ laser cutter. Before conducting the drainage experiments, the fractures were saturated by injecting ink-dyed distilled water. After that, the fluid was withdrawn at a constant flow rate (0.1 mL/min) under atmospheric conditions. During the drainage process, an optical imaging system (similar to that described in Section 3.1.1) was used for capturing high-resolution images of the air-water interface. These images were used to calculate the interfacial velocities at different times of the fluid drainage along with the fractures. The calculations indicated that the interfacial velocities represent significant Haines jumps when the meniscus passes from a narrow throat to a wide body. As stated by Chang et al. [317], this finding may help in understanding the origin of interface instabilities and the resulting non-uniform phase distribution, as well as the micron-scale essence of the spatial and temporal instability of two-phase flow in fractured media at the macroscopic scale. 

Hsu et al. [15] used the COC micromodel in a series of two-phase flow experiments involving imbibition and drainage processes in fractured porous media. The aim of these experiments was to investigate the spatial distribution of oil and water when the fluids were subjected to various injection and extraction rates; 0.83, 1.67, 3.33, and 6.67 mm^3^/s (50, 100, 200, and 400 μL/min). The spatial distribution of fluids was captured by using a high-speed camera (frame rate of up to 150 fps). In general, the experimental results showed that the water- and oil-extraction efficiency in a fractured porous medium depends on the boundary conditions, the injection and extraction rates, and the dimension of a fracture. Although these experiments did not confirm the common assumption that the fluids such as water and oil are extracted first from a large fracture rather than from small pores, they provided evidence of the existence of a new residual trapping mechanism in porous media during the drainage and imbibition processes [15].

### 4.3. Reactive Transport, Solute and Colloid Transport in Porous Media

A thorough understanding of reactive transport and solute mixing in porous media is critical for optimizing and managing different engineered and natural processes. The quantification of the relative importance of diffusion, advection, and dispersion on reactions and mixing in geological formations is immensely challenging. Due to the complexity and heterogeneity of porous media, it is not trivial predicting the location and rate of reactions occurring in the media [319]. Moreover, the precipitation and dissolution of minerals affect porous media porosity, permeability and surface area which lead to a dynamic relationship between transport and reactive processes. Therefore, it is extremely important to visualize these processes to obtain an in-depth understanding.

#### 4.3.1. Solute Transport 

Corapcioglu et al. [9] used a glass micromodel with a regular geometry of orthogonal channels to study and model solute transport in porous media at pore-scale. To observe the solute transport and obtain the concentration contours of the solute front, they injected dye solutions as tracers at a constant flow rate, video recorded the process and analyzed the images. Glass micromodels consisting of complex flow network patterns were also used for examining the migration of organic fluid (a mixture of oil and Soltrol-130^®^ solvent) through a saturated aqueous zone, as well as for investigating the dissolution of organic liquid in the water-saturated porous media [101]. 

Sato et al. [320] visualized the process of CO_2_ migration and trapping in a Berea sandstone rock sample using X-ray CT. They injected one pore volume (PV) of CO_2_ into a saturated core sample with water and then successive water injections were performed. They found that after 15 PV water injection, the CO_2_ saturation was decreased from 30% to 10%. Moreover, 2D and 3D neutron imaging were used by Cordonnier et al. [321] to study cadmium (a common contaminant in soil and groundwater) sorption and transport in porous media. They performed a series of flow-through experiments on different sandstone and limestone core samples and imaged in-situ flow properties. NMR measurements were used by Colbourne et al. [322] to observe the temporal evolution of the reactive flow of an acid in a rock sample. They investigated the effect of wormhole formation on fluid displacement efficiency. Richter et al. [323] applied PET imaging for geochemical modelling in an unsaturated clay. Robust and reliable geochemical models are required to simulate and predict water flow for a long-time secure storage. They visualized the front progress and distribution of tracer concentration and by using the measured flow profiles and demonstrated that an advective-dispersive mechanism governs solution transport in unsaturated clay.

Recently, Pini et al. [324] applied PET and X-ray CT for a better understanding of solute spreading and mixing in Berea sandstone core samples. The high spatial (millimetres) and temporal (tens of sec) resolution of PET helped them to visualize the spatial and temporal evolution of the solute (tracer) plume at the core scale. The dual-energy Gamma radiation technique has been used only for a few solute transport studies with a focus on breakthrough time. Oostrom et al. [325] used the gamma radiation technique to determine in-situ salt concentrations and its local longitudinal dispersivities in saturated soil columns. They performed a series of displacement experiments with deionized water and a NaI solution and derived longitudinal dispersivities values from in-situ concentration break- through curves using gamma radiation. They found that the dipersivity values obtained from this technique are smaller than those derived from conventional technique using effluent concentration breakthrough curves.

#### 4.3.2. Effect of Pore-scale Heterogeneity 

Zhang et al. [326] investigated the effects of pore-scale heterogeneity on transverse mixing on the growth, distribution, and activity of biomass in porous media for bioremediation purposes. They fabricated two silicon-Pyrex micromodels with different degrees of heterogeneity using the technique presented by Chomsurin and Werth [327]. One micromodel with a uniform array of cylindrical pillars as the homogeneous system and another micromodel with clusters of large and small cylindrical pillars as the heterogeneous system were used in their study. They acquired micromodel images using an epi-fluorescent micro-interference contrast (DIC) microscopy. They observed more uniform and rapid biomass growth and more degradation in the homogeneous micromodel than the heterogeneous one.

Van Offenwert et al. [221] studied effect of pore-scale heterogeneity on solute spreading and mixing. They developed a novel methodology using fast laboratory-based micro-CT system to quantify transient solute concentration fields at pore-scale. With a time resolution of 15 s and a spatial resolution of 13.4 μm, they could dynamically capture 3D images of injecting a tracer into two samples (sandstone and sintered glass) with different level of heterogeneity. Their results showed greater dispersion in the sandstone sample with more heterogeneity than sintered glass.

#### 4.3.3. Dissolution & Precipitation

Kim et al. [328] developed a PMMA micromodel to study pore-scale salt precipitation during CO_2_ storage in saline aquifers. They observed two types of salt formations (large bulk crystals and polycrystalline aggregated structures) which resulted in a significant reduction in the porosity of porous media due to salt precipitation.

**Figure 8:**
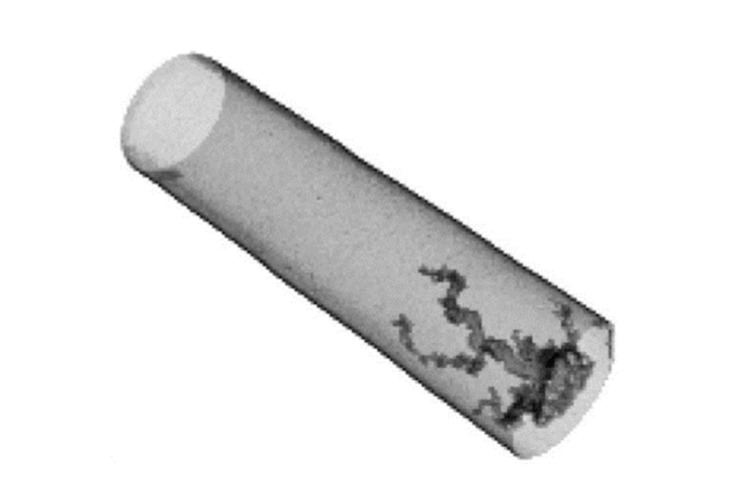
Neutron computed tomography for dissolution of the carbonate by the CO_2_-rich brine (Reprinted with permission from [233]).

Cai et al. [221] utilized a flow-column and X-ray µCT to study dissolution and precipitation processes in pore structures exposed to simulated caustic waste. The flow-column was 8.8 cm in height and imaging considerations (e.g., 4 µm voxel size and beam energy) meant that a small inner diameter of 3.1 mm was used. Stacks of images were taken along the length of the flow-column and 3D images were reconstructed. They quantified the reduction in porosity due to precipitation and demonstrated that the large pores were dominated by dissolution, while small pores were affected by precipitation. Figure 8 shows visualization of dissolution of carbonate (limestone) core by CO_2_-rich brine flooding experiment using neutron computed tomography [233]. Bray et al. [329] utilized X-ray μCT and MRI techniques in a series of experiments to observe precipitation of CaCO_3_ mineral a porous media and investigate its effect of fluid flow. They performed a parallel injection of Na_2_CO_3_ and CaCl_2_ under two relative flow rates in a flow cell packed borosilicate glass microsphere (180–212 μm) and saturated with deionized water. They found that precipitation can minimize dispersive and advective transport between the two fluids. 

#### 4.3.4. Colloids Transport 

Colloids are small particles with typical size ranges from a few nanometres to one to ten micrometres which are usually suspended in a solution. Processes such as filtration, groundwater contamination and waterflooding for oil recovery are examples of colloidal dispersions flow in porous media. Deposition of colloids in a porous media can significantly modify porosity and permeability of medium [330]. Auset and Keller [16] used PDMS models to investigate the effect of the size of colloids (particles) and pores on colloidal dispersion in porous media. In these experiments, colloids of different diameters (carboxylate-modified latex polystyrene microspheres with φ = 2, 3, 5, or 7 μm) were injected and transported through the pore networks of three different models under four different differential pressures; 0.1, 0.5, 1, and 1.5 kPa (ΔP = 1, 5, 10, and 15 mbar). The particle trajectories, residence times, and dispersion coefficients through the models were determined by using an image analysis software. In general, these experiments provided evidence that the magnitude of the dispersion at any given flow rate is controlled by the pore-space geometry and the relative size of colloids with regards to pore channels. Zhang et al. [331] used a PDMS micromodel combined with confocal laser scan microscopy (CLSM) imaging technique to investigate colloids interaction with liquid phases, liquid-liquid interfaces and liquid-solid interfaces. They fabricated a closed PDMS micromodel with uniform and stable hydrophobic wettability conditions. Moreover, they used a very thin glass substrate coated with a film of PDMS to seal the model so they could focus at locations throughout the whole depth of micromodel. Using CLSM technique the movement of fluorescent particles (300 µm in diameter) and flow of two liquids within the porous structure of micromodel were visualized for better understanding of colloids removal process. Seiphoori et al. [332] also investigated the assembly of aggregates formed by evaporating various suspensions using a PDMS microfluidic device containing a single channel.

Baumann and Werth [333] performed experimental and numerical simulation studies on colloid transport in porous media. Flow paths and particle velocities for different water injection rates in a silicon-Pyrex micromodel with cylindrical pillars were captured using epifluorescent microscopy. They compared experimental results with simulation results of a 2D lattice Boltzmann (LB) model. Colloidal deposition in porous media was studied by Gharbi et al. [334] using a gamma ray technique. Using this imaging technique enabled them to measure the local deposition and variation in the porosity. 

### 4.4. Porous Media (Rock) Characterization & Rock/Soil Deformation

The characterization of porous media is critical for estimating essential parameters such as porosity, pore size distribution, permeability (conductivity), wettability, and other flow functions. These parameters are pertinent input for modelling and numerical simulation of different processes in porous media from pore-scale to field-scale. Therefore, the accuracy and reliability of numerical simulation results are heavily reliant on the characterization techniques.

#### 4.4.1. Porosity and Pore Size Distribution 

Neutron tomography was used by Kichanov et al. [335] to obtain volumes, size distribution, and orientation distribution of mineral grains for rock samples to study the origin of the crust. Xiong et al. [272] investigated applications of NMR measurements in obtaining pore-size distribution of a porous material by measuring the transverse relaxation time. NMR relaxometry has a short measurement time for pore size distributions. However, any induced error in transverse relaxation times or unwanted effects on a multimodal relaxation time distribution function can lead to erroneous calculated pore size distributions. Gamma rays were used to measure the porosities of several core samples having different lithology and range of porosities [336]. It was found that small scale heterogeneities can be characterized using gamma radiation and provide more insight into the type of porosity.

#### 4.4.2. Hydraulic Conductivity 

Recently, Gueven et al. [337] have examined several different polydispersed sintered glass bead systems with the use of X-ray computed tomography (this visualization technique is described in Section 3.2.1) and evaluated the impact of the sintering procedure and the original particle size distribution on the hydraulic properties of the models. The experiments demonstrated that the intrinsic permeability in sintered granular packings depends not only on the porosity, but also the size of pore throats. Although future work is needed to extend this study towards systems with a lower porosity (<32%), the reported findings are useful to evaluate the hydraulic characteristics of many porous systems (e.g., natural rocks like sandstone). Schmitt et al. [212] developed a methodology to classify and quantify the shape of irregular rock pore/particles using 3D X-ray images. They visualized the main pore networks and several disconnected pore ganglia for three sandstone samples. Moreover, 3D imaging has been used for pore network extraction and many methods for network extraction have been proposed and subsequently used for pore-scale modelling [12]. Hicks et al. [219] proposed a method for measuring core porosity and residual oil saturation in a heterogeneous carbonate core sample using X-ray CT. They obtained average saturation on the millimetre scale, evaluated core heterogeneity and presented a relationship between porosity and residual saturation. NMR measurements were used to estimate the permeability of a water-saturated sandstone cores with diameter of 2 cm and length of 3.75 cm [338].

Understanding physical and chemical processes in geosciences at nanoscale is of increasingly interest. Analytical TEM has much higher magnification than other methods such as scanning electron microscopy (SEM, Section 3.3.2), and therefore, acquiring more information at pore scales [339]. Curtis et al. [275] investigated the pore connectivity and flow paths of shale samples to understand the governing mechanisms of gas production from shale reservoirs. They employed TEM and SEM techniques to visualize pores with diameter size of less than 3 nm of 125 µm^3^ shale sample. They combined these techniques with mercury injection capillary pressure (MICP) and obtained very useful information about the pore structure, pore connectivity and mechanical properties of the shale sample [275].

#### 4.4.3. Wettability 

Müehl et al. [340] used confocal laser scanning microscopy (CLSM) to visualize the area and connectivity of the water and the thickness of water films in silica sand samples with different wetting properties. They altered wettability of silica sand by silanization, making the sample surface less hydrophilic (contact angle less between 0° and 90°). A segmentation strategy was developed to separate water films and bulk water during image processing. They found CLSM a very useful visualization tool to study effect of wettability on water configuration in porous media. Being able to visualize water focused over the pore space is one great advantage of CLSM over conventional microscopy. CLSM is also a simpler 3D imaging technique compared to others. 

NMRI has been used to investigate rock wettability as one of the crucial parameters in recovery factor of oil and gas reservoirs. Conventional approaches, such as Amott and U.S. Bureau of Mines (USBM) methods, for investigating reservoir wettability may not be applicable for shales due to low permeability, complex pore structure, variation in mineralogy and organic constituents. Odusina et al. [341] used NMRI to study wettability of shale samples. They used Berea sandstone as a reference and analysed 50 shale samples from four different reservoirs, showing mixed wettability for the studied samples. They also estimated the width of fractures in range of 1 to 10 μm which was compatible with the images from X-ray µCT.

#### 4.4.4. Multi-Scale Heterogeneity 

Shah et al. [342] presented an improved sample-preparation technique for imaging of challenging porous materials such as carbonate rocks using CLSM. Carbonate rocks are heterogeneous with complex pore structures and microporosity, and an appropriate sample preparation can help CLSM to capture this complexity. 

Chen et al. [274] presented a workflow for integrating different imaging techniques to study heterogeneity in shales. Their objective was to use multiscale imaging and numerical simulation to estimate macroscale properties using measured micro-scale parameters. They integrated µCT, 2D SEM (mm field of view), TEM and FIB-SEM techniques with numerical simulation to calculate porosity, permeability and two-phase relative permeability for a shale rock sample [274]. Interestingly, FIB-SEM imaging has been used as a calibration tool for wireline well logs analysis. By employing the FIB-SEM images, Ahmad and Haghighi [343] obtained more information about the level of heterogeneity, type of existing porosities, brine and organic matters for an Australian shale gas reservoir. They evaluated different petrophysical models for calculating the shale content, porosity and water saturation and found the most applicable models. Recently, Li et al. [213] combined FIB-SEM and X-ray µCT to quantitatively characterize pore-fracture networks in coals at different scales. They built a pore network model using the information obtained for size of pores and throats from image analysis and investigated the mechanisms of coalbed methane (CBM) storage. After carefully reviewing different techniques for pore structure characterization in tight sandstones, they suggested utilizing combinations of several measurements including SEM, X-ray µCT, MICP, and NMR [344].

#### 4.4.5. Rock and Soil Deformation 

Deformation of the internal structure of porous media through a variety of natural and engineering processes affects soil and rock properties. Dissolution and biological activities result in gradual deformation however swelling/shrinking and landslides can cause abrupt deformation. These deformations processes alter porosity, permeability and mechanical properties of rock or soil. Application of imaging techniques for understanding deformation processes and rock and soil characterization significantly improved conventional methods which only provide limited properties, such as bulk density and porosity [220].

Schlüter et al. [220] used X-ray CT to capture deformation of soil structure due to high angular velocity when measuring capillary pressure via centrifuge. They investigated the soil deformation for two rock samples with different texture and content and found that shrinkage and compactions are two main causes. Tudisco et al. [345] studied deformation of a Bentheim sandstone core sample and utilized neutron tomography to acquire images from internal structure of the sample. They applied 3D volumetric digital image correlation (3D-DIC) using pre- and post-deformation images to measure deformation and map 3D localized strain fields. They compared the results of neutron imaging against X-ray and concluded that images from neutron tomography can be used for mechanical analysis through 3D-DIC. However, their resolution is less for the same voxel size. To perform in-situ triaxial tests on a rock sample, they had to apply 40 MPa (400 bar) confining pressure. Therefore, they needed a metal pressure vessel with thick walls and to tackle the challenge of penetration depth for imaging, neutron was superior to X-ray [257,345].

Tudisco et al. [346] also used neutron radiography in another set of experiments to track fluid front, while injecting a fluid in the deformed sandstone sample. They used neutron radiography to be able to follow the fluid flow which is a fast process in a deformed media with possible fractures. A good agreement between flow measurements and strain fields was observed. The images from fluid flow showed higher front progression in the deformed zone which can be evidence of higher permeability and porosity. Charalampidou et al. [347] investigated the interaction between pure and shear-enhanced compaction bands and fluid flow in porous media in a water imbibition process by using in-situ high speed neutron tomography (1 min per tomography). As neutron imaging is sensitive to hydrogen, they had a high image contrast between water and rock material and could visualize the waterfront during the imbibition process. The results showed that the induced compaction bands affect the porosity, permeability, and propagation of flow front significantly and should be thoroughly investigated in different applications such as geological CO_2_ storage [347].

## 5. Summary and Final Remarks

The aim of this paper was to critically review both micromodels and imaging methods to provide the community with the most recent advances in visualization techniques in porous media and their applications in geoscience and geo-energy engineering. 

This review analysed fabrication methods for micromodels, particularly focusing on the replication of the internal structure of geomaterials. Moreover, the chemical, mechanical and thermal properties of micromodels should also satisfy the requirements of experimental studies. Fabrication techniques have significantly improved over the decades in which they have pushed the boundaries of features’ dimensions down to a few microns. The review covered glass-based, photoresist-based, polymer-based, silicon-based, and hybrid geomaterial-based micromodels. 

The fabrication of glass-based and silicon-glass-based micromodels is a multi-step process that requires the use of a specialized equipment, exposure masks, hazardous chemicals, and a clean room. Therefore, the whole fabrication process of this type of micromodels can be expensive and time consuming. In contrast, industrial lasers have become very popular tools in modern manufacturing due to their flexibility, controllability, and ability to process in three dimensions. Recently, for instance, it has been shown that selective laser etching (SLE) process enables the manufacturing of 3D microfluidic devices in glass [93]. They found that SLE is a suitable process for the mass production of 3D structures as a faster writing speed showed higher selectivity and higher precision of the resulting structures. Although the channels manufactured in this way are still at least an order of magnitude larger than the pores and throats in real geomaterials, this process shows potential to become an effective process in the manufacturing of pore network micromodels. Another promising method for the fabrication of enclosed pore network micromodels using glass substrates has been recently developed [94,95]. This method uses an ultrashort pulse laser both for the generation of the network of pores and micro-channels by laser ablation, followed by bonding of glass plates together by laser micro-welding. 

Development of geomaterial micromodels via either combining existing fabrication methods with mineral coating techniques or using minerals as part of micromodel materials has improved significantly. Micromodels with more realistic surface chemistry, roughness, and wettability are currently receiving significant interest for research in fluid flow and reactive transport. Integrated lab-on-chip apparatus and experimental set-up where one or more optical imaging techniques, e.g., micro-PIV or Raman microscopy are combined with geomaterial micromodels are of broad and current interest. Invaluable in-situ measured data such as velocity fields, shear stress at the fluid/fluid interface, dissolution and precipitation of minerals will profoundly contribute to validation and refinement of numerical models of multi-phase flow and reactive transport.

The application of 3D printing in geoscience is growing rapidly and significant progress has been made towards creating rock replicas. However, more improvements are required in terms of having a better control on surface roughness and its distribution in printed samples. Minimum printing size of features both in micromodels and rock replicas has reached down to microns, but the challenge of removing support material needs to be resolved at this scale.

This review also analysed imaging techniques used to understand a variety of complex processes in porous media. In geosciences and petroleum engineering, porous media characterization (pore size, shape, structure, connectivity, and distribution), multiphase fluid distribution, fluid flow mechanisms, solute transport, and reactions have been studied using these techniques. In general, optical imaging techniques are the most common and easiest options for visualization of fluid flow and reactive transport in micromodels. µPIV is an optical and nonintrusive technique that has provided 2D or even 3D velocity field micron scale for flow test in microfluidic devices which has had significant contribution in the process of verification of mathematical models and numerical simulations. X-ray CT, a technique with a resolution down to a few hundred nanometres, has gained significant interest for temporal and 3D spatial monitoring of different processes in porous media. Despite all the advantages of X-ray CT, its image analysis is operator dependent due to discretization effects and imaging artifacts. Moreover, 4D CT has made visualization of dynamic process possible which has been a remarkable improvement in imaging industry. Currently, this technique has gained a significant attention due to its wide range of applications. The application of artificial intelligence algorithms in image processing and establishing standard workflows is going to be a research trend in this area. For instance, developing automated techniques for in-situ and dynamic contact angle measurements at pore-scale is a subject undergoing intense study.

The principles of neutron tomography are the same as X-ray CT, but neutrons have a greater depth of penetration than X-rays and can track flow fronts. NMRI has been applied to 1D, 2D and 3D imaging of different fluid flow and reactive transport processes in porous media but this is costly due to the high capital cost. Integration of PET and X-ray CT and positron emission particle tracking have gained interest geoscience research areas. The study scales of microscopy methods are smaller than representative elementary volume, but information about pore structure and connectivity can be obtained to improve our understanding of fluid flow processes. In general, integration of X-ray CT, 2D SEM (mm field of view), TEM and FIB-SEM techniques improves the characterization of a complex rock sample and is very useful for pore-scale modelling purposes. Continuing improvements are being made in reducing data acquisition time and cost. Today, fast lab-based synchrotron X-ray µCT is more accessible which allows us to visualize and quantify pore-to-pore displacement during fluid flow, which sheds light on trapping mechanisms in EOR, CO_2_ sequestrations and other processes. 

Although by using imaging techniques valuable information such as saturation profiles or fluid fronts can be obtained, there is still a need for quantitative, in-situ and dynamic measurements for model validation purposes. This time-dependent information is important to understand the role of different active parameters and forces thoroughly and validate models of pore-scale displacement. Knowledge of in-situ contact angles obtained by X-ray µCT imaging can now be feed into pore-scale models, e.g., pore network models, for a more reliable prediction of relative permeability and capillary pressure curves. Embedded fibre optic sensors inside a microfluidic device for measuring pressure, temperature, pH, and other flow properties can be a substantial step toward the refinement of mathematical models. Recently, Zarikos et al. [305] demonstrated a PDMS micromodel with embedded fibre optic pressure sensors to measure pressure at the pore-scale for single- and two-phase (drainage and imbibition) processes. 

Characterizing porous media, extracting pore structures, and visualizing flow using micromodel testing and high-resolution imaging techniques provide a powerful platform for developing realistic and accurate mathematical models, which can be used as a predictive tool for fluid flow in porous geomaterials. Incorporating time-dependent in-situ measurements in order to refine mathematical models is an important research area to further advance the field of fluid flow studies in porous media.

## Figures and Tables

**Table 1:** Different types of micromodels reviewed in this paper.

Type of Models		Pattern Generation Methods	Bonding Techniques	Advantages	Limitations	Selected Applications
Glass-based micromodels	Glass bead	Arranging of glass beads in transparent container	■ Thermal bonding ■ Adhesives	■ Simple and inexpensive fabrication process ■ Models suitable for high-pressure experiments	■ Limited number of pore network patterns ■ Challenging to replicate an internal structure of real rocks ■ Optical visualization of pores may be obstructed ■ Problem with the observation of flow phenomena in more than 1 layer of glass	■ Fluid flow processes ■ Fingering effects caused by fluids of two different viscosities in inhomogeneous porous structures ■ Dissolution of solvents and compounds in porous media
	Glass plates	■ Dry etching (RIE) ■ Wet etching ■ Direct laser writing Selective laser etching (SLE) process	■ Thermal bonding ■ Laser welding	■ Highly-transparent, chemically resistant and thermally stable ■ Micromodels suitable for high-pressure experiments ■ Ability to generate 3D micromodels by using SLE	■ Expensive to fabricate in small quantities by dry etching (RIE) ■ Chemical hazard and high disposal costs (wet etching) ■ SLE is a slow process	■ Fluid displacement ■ Enhanced oil recovery ■ Transport of colloids ■ Dissolution of compounds
Photoresist-based micromodels		Photolithography	Pressing cover glass to soft photoresist, then hard baking	■ Well-established fabrication process ■ Simple bonding process ■ Pores can be < 1 μm	■ Micromodels unsuitable for high-pressure applications ■ Gradual degradation of photoresist in time	■ Fingering effects ■ Fluid displacement
Polymer-based micromodels	PDMS	Soft lithography	■ Pressing (reversible) ■ Plasma treatment, corona discharge, PDMS curing (irreversible)	■ Relatively inexpensive and well-established fabrication process ■ Fast fabrication and low cost ■ Ability to generate 3D micromodels	■ Deformation of channels and pores even at low pressure ■ Incompatible with organic solutions ■ Unstable wetting properties of PDMS in time	■ Fluid displacement ■ Transport of colloids
	PMMA	■ LIGA process ■ Direct laser writing	■ Thermal bonding ■ Adhesives	■ Highly-transparent ■ High stiffness ■ Direct laser writing enables rapid prototyping of micromodels	■ Expensive to fabricate in small quantities (by LIGA process – see Section 2.2.2 below) ■ Laser-generated structures have imperfections	■ Fluid displacement ■ Fluid flow through fractures
	COC	Photolithography & hot embossing	■ Adhesives ■ UV treatment ■ Ozone treatment	■ Highly-transparent ■ High stiffness ■ Resistant to acids & solvents	■ Expensive to fabricate in small quantities ■ Pore dimensions > 100μm	■ Fluid flow (drainage and imbibition) through fractures
	Resin-based 3D printing	3D printing technology (layer by layer fabrication)	■ Adhesives ■ UV treatment	■ Relatively fast and low cost ■ Micro-scale micromodels and Darcy-scale rock sample replica	■ Further development required ■ Unwanted porosity ■ Different resolution in XYZ directions	■ Fluid displacement ■ Reactive transport
Silicon-based micromodels		■ Dry etching (Bosch process) ■ Chemical etching	Anodic bonding	■ Possible to replicate internal structures of rocks ■ Models suitable for high-pressure experiments	■ Expensive to fabricate in small quantities ■ Optical access limited to one side only.	■ Fluid displacement ■ Fingering effects ■ Trapping
Hybrid geomaterial-based micromodels		■ Selection of above techniques ■ Mineral coating	■ Selection of above techniques	■ More real representative of rock samples ■ Including minerals ■ More realistic wettability conditions	■ Visualization due to 2.5 D/3D nature ■ Less control on distribution of pores ■ Repeatability of fabricated samples	■ Fluid displacement ■ Reactive transport ■ Wettability distribution ■ Surface roughness

**Table 2:** Summary and comparison of imaging techniques reviewed in this paper.

Imaging Technique	Image Dimensions	Image Resolution (IR)	Advantages	Limitations	Selected Applications
Optical Imaging—camera & microscope-camera	2D	few μm (>1 μm)	non-invasive non-destructive	Not suitable for 3D models Relatively low resolution (camera only)	saturation distribution fluid flow mechanisms reactive transport particle dynamic
Optical Imaging—Photoluminescent volumetric imaging (PVI)	3D	submicron (<μm)	non-invasive non-destructive high resolution capturing very slow processes	relatively expensive limited effective depth of penetration	saturation distribution fluid flow mechanisms reactive transport particle dynamic
Optical Imaging—Raman Microscopy	3D	submicron (<μm)	non-invasive non-destructive high resolution small sample volume not interfered by water	cannot be used for metals or alloys sample damage due to heating caused by laser high cost	reactive transport mineral characterization characterization of molecular structures
Optical Imaging—Micro Particle Image Velocimetry (µPIV)	2D/3D	down to few μm (1–10 μm) for length scale of 100 μm	non-invasive non-destructive high resolution	high-speed flows near surfaces measurements difficulty of homogeneous particle seeding	fluid flow in-situ measurements velocity fields
X-ray Computed Tomography	3D	few μm to Darcy scale * medical: 200–500 μm industrial: 50–100 μm synchrotron: 1–50 μm Temporal: ~30 min to 10 s	non-invasive non-destructive 4D (spatial & temporal)	operator dependency discretization effects imaging artefacts	pore space characterization saturation distribution fluid flow mechanisms deformation of materials reactive transport pore scale modelling
Neutron Tomography	3D	16–100 μm (FOV: 33–205 mm) Sample size of 100 cm3 volume at medium resolution	non-invasive non-destructive high penetration depth front visualization large samples	operator dependent discretization effects imaging artefacts less resolution than X-ray longer acquisition time than X-ray less available	saturation distribution deformation multi-phase flow distribution of organic and inorganic carbon
Positron Emission Tomography (PET)	3D	clinical: 3–5 mm biomedical: 1 mm high temporal resolution: 10 s	non-invasive non-destructive combined with CT high depth of penetration	not a mature technique	fluid distribution microbial processes geochemical studies
Nuclear magnetic resonance (NMR) & Magnetic Resonance Imaging (MRI)	3D	tens of μm for cm-samples mm’s for m-samples	non-invasive non-destructive	Expensive Long acquisition time (in comparison to XCT)	pore size distributions fluid distribution reactive transport wettability
Dual-Energy Gamma Radiation	2D	cm scale (Darcy scale)	non-invasive non-destructive large scale imaging	long imaging time average (global) values (e.g., average saturation)	porosity dry bulk density saturation distribution
Transmission Electron tomography (TEM)	2D	down to few nm for 100 µm^3^ sample size	non-invasive non-destructive high resolution	Small FOV (not suitable for Darcy scale)	shale porosity wettability pores connectivity pore geochemistry
Focused Ion Beams Scanning Electron Microscopy (FIB-SEM)	3D	down to few nm	non-invasive high resolution	destructive (FIB) not suitable for Darcy scale not appropriate for heterogeneous samples	shale porosity wettability pores connectivity

* Darcy scale: at this scale, each spatial point contains large number of pores, occupied by multiple fluid phases and each phase forms a continuum over the entire spatial domain [148].

**Table 3:** Summary of different applications of micromodels and imaging technique in geoscience and geo-energy engineering reported in this paper.

Type of Models	Micromodels							
	Photoresist-Based	Polymer-Based			Glass-Based		Silica-Based	Geo-Material
		PDMS	PMMA	3D Printing	Glass Beads	Glass Plates and Hele Shaw		
Fluid displacement (Drainage & Imbibition), Single- and multi-phase flow mechanisms, Gravity drainage, Capillary rise, Infiltration, Flow instability (e.g., viscous fingering), Saturation distribution, Trapping/residual saturations	Oxaal (1991), Cheng & Giordano (2002)	Qi et al. (2009), Qin et al. (2010), Wu et al. (2012), Karadimitriou et al. (2013), Xu et al. (2014), Watson et al. (2018)	Hsu et al. (2017), Chang et al. (2017), Tsakiroglou and Avraam (2002), Chapman et al. (2013), Hsu et al. (2017), Ju et al. (2017)	Watson et al. (2018)	Chatenever & Calhoun (1952), Saffman & Taylor (1958), Chuoke et al. (1959), Lu et al. (1994a, 1994b, 1995), Manz et al. (1999a, 1999b), Nguyen & Miller (1993); Cinar et al. (2009), Lu et al. (2018)	Wardlaw (1982), Sohrabi et al. (2004, 2008; 2008, 2017), van Dijke et al., (2006), Riazi et al. (2011), Keller et al. (1997), Bijeljic et al. (2001)	Buchgraber et al. (2012), Bandara et al. (2013), Wang et al. (2012), Li et al. (2017), Dimou et al. (2019), Watson et al. (2018), Kazemifar et al. (2015)	Song et al. (2014), Song & Kovscek (2015), Song & Kovscek (2016), Zhu & Papadopoulos (2012), Bowden et al. (2016), Tanino et al. (2017 & 2018), Wang et al. (2017), Alzahid et al. (2018), Porter et al. (2015), Bowden et al. (2016)
Fractured rocks and heterogeneous media	Cheng et al. (2004)	Qi et al. (2009), Qin et al. (2010)	Chang et al., (2017), Ju et al. (2017), Yu et al., (2019)	Suzuki et al. (2017), Ahkami et al. (2019)	Karambeigi et al. (2013)	Keller et al (1997), Corapcioglu et al. (1997), Rangel-German & Kovscek (2006), Bijeljic et al. (2001), Wan et al. (1996), Farzaneh et al. (2010), Kamari et al. (2011)	Oostrom et al. (2016), Roman et al. (2016), Rangel-German & Kovscek (2006), Chomsurin & Werth (2003), Zhang et al. (2010)	Porter et al. (2015), Gerami et al. (2017), Bowden et al. (2016), Alzahid et al. (2018)
Reactive transport, Transport of colloids, solute and particles, microbial treatment	-	Singh et al. (2017), Soulaine et al (2017), Auset & Keller, (2004), Zhang et al. (2013)	Kim et al. (2013)	Ishutov et al. (2017, 2018a, 2018b), Kitson et al. (2012)	Karambeigi et al. (2013)	Conrad et al. (1992), Danesh et al. (1988), Corapcioglu et al. (1997), Doryani et al. (2016), Goldenberg et al. (1989) Wan & Wilson (1994)	Zhang et al. (2010), Oostrom et al. (2016), Chomsurin & Werth (2003), Baumann & Werth (2010)	Song et al (2014), Song & Kovscek (2016), Singh et al. (2017)
Velocity profile (local & field)	-	Heshmati and Piri (2018)	Chang et al., (2017), Ju et al. (2017), Yu et al., (2019)	Ahkami et al. (2019)	Al-Mugheiry et al. (2001), Lu et al. (2018)	Bijeljic et al. (2001)	Roman et al. (2016)	-
Porous media characterization, wettability effect, Rock/soil deformation	Cheng & Giordano (2002)	Schneider & Tabeling (2011)	-	Kong et al. (2019a, & 2019b), Ishutov et al. (2017, 2018a, 2018b), Head & Vanorio (2016),	Gueven et al. (2017)	Lee, et al. (2015), Morrow et al. (1986) Wardlaw (1982), R. Hu et al., (2017), Lee et al. (2015)	-	Ishutov et al. (2017), Song et al (2014), Song and Kovscek (2015), Gerami et al. (2017), Song and Kovscek (2016), Tanino et al. (2017 & 2018), Wang et al. (2017), Alzahid et al. (2018)
Fluid displacement (Drainage & Imbibition), Single- and multi-phase flow mechanisms, Gravity drainage, Capillary rise, Infiltration, Flow instability (e.g., viscous fingering), Saturation distribution, Trapping/residual saturations	Li et al. (2017), Kazemifar et al. (2015)	-	Wang et al (1984), Hicks et al. (1992), Kumar et al. (2009), Oughanem et al. (2013 & 2015), Sato et al. (2012), Al-Menhali et al. (2015), Oughanem et al. (2013 & 2015)	Charalampidou et al. (2017), Cordonnier et al. (2019)	Manz et al. (1999a, 1999b), Dijk & Berkowitz (1999), Al-Mugheiry et al. (2001), Bijeljic et al. (2001), Colbourne et al. (2016)	Khalili et al. (1998), Haugan (2000), Boutchko et al. (2012), Hu et al. (2017)	Nicholls & Heaviside (1988), Huang & Gryte (1988), Ursin (1992), Oostrom et al. (2003), Brusseau et al. (2008), Cihan (2008)	-
Fractured rocks and heterogeneous media	Ahkami et al. (2019), Yu et al., (2019)	-	Hicks et al. (1992), Howard et al. (1993), Van Geet & Swennen (2001), Brattekas et al. (2016), Schmitt et al. (2016)	Lewis et al. (2017), Tudisco et al. (2015),	Manz et al. (1999a, 1999b), Dijk & Berkowitz (1999)	Kulenkampff et al. (2015 & 2016), Brattekas et al. (2016)	Ursin (1992)	Chen et al. (2013), Ahmad & Haghighi (2013), Li et al. (2017),
Reactive transport, Transport of colloids, solute and particles, microbial treatment	-	Singh et al. (2015, 2017), Poonoosamy et al. (2020)	Richter et al. (2005), Wilding et al. (2005), Cai et al. (2009), Bray et al. (2017), Brattekas et al. (2016)	Cordonnier et al. (2019)	Colbourne et al. (2016)	Kulenkampff et al. (2015 & 2016), Brattekas et al. (2016), Kinsella et al. (2012), Pini et al. (2016)	Brusseau et al. (2008), Oostrom et al. (1992), Gharbi et al. (2004)	-
Velocity profile (local & field)	Ahkami et al. (2019), Lu et al. (2018), Roman et al. (2016), Kazemifar et al. (2015), Yu et al., (2019), Heshmati and Piri (2018)	-	-	-	Al-Mugheiry et al. (2001), Bijeljic et al. (2001), Dijk & Berkowitz (1999)	Hu et al. (2017)	-	-
Porous media characterization, wettability effect, Rock/soil deformation	-	Singh et al. (2015, 2017), Poonoosamy et al. (2020)	Gueven et al. (2017), Hicks et al. (1992), Head & Vanorio (2016), Al-Menhali et al. (2015), Schluter et al. (2016), Charalampidou et al. (2013), Tudisco et al. (2015)	Kichanov et al. (2015), Tudisco et al. (2015), Cordonnier et al. (2019)	Xiong et al. (2016), Odusina et al. (2011)	-	Bodwadkar & Reis (1993)	Curtis et al. (2011), Wu & Aguilera (2012), Chen et al. (2013), Ahmad & Haghighi (2013), Li et al. (2017)

## Glossary

Anodic bonding	A process to seal silicon and glass which involves heating and applying an electrical field.
Capillary pressure	The pressure difference between two immiscible fluids across the interface between two static fluids
Creeping flow	When the Reynolds number is very small (<<1) where the viscous forces of the fluid dominate the inertial forces.
Darcy’s law	An empirical equation that describes the flow of one fluid in a porous media.
Delaunay triangulation (in computational geometry)	A triangulation of a given set of vertices such that no vertex in the set is inside the circumcircle of any triangle in the triangulation.
Drainage	The displacement of a wetting fluid (phase) by a non-wetting fluid (phase).
Enhanced oil recovery	The process of increasing the recovery of oil (hydrocarbon) from an oil reservoir e.g, by injecting a miscible gas.
Etch selectivity	The ratio of the etch rate of the unprotected layer to the etch rate of the layer on the projection mask.
Forchheimer equation	Darcy’s law is only valid for slow fluid flow in porous media and for flows with Reynolds numbers greater than about 1 to 10, Forchheimer proposed an equation to account for the non-linear effect of turbulence flow on pressure drop.
Geo-material	Any material with geological origin, e.g., rocks.
Haines jump	Are sudden jumps of the fluid interface in pores which promote fluid redistribution, fingered invasion and fluid trapping in pore-scale.
Imbibition	The displacement of a non-wetting fluid (phase) by a wetting fluid (phase).
Laminar flow	When a fluid flows in parallel layers and there is no disruption between the layers. For fluid flow in pipes, laminar flow happens when Re < 2300.
Micro-scale	In the scale of micrometre. Interchangeably is used with pore-scale.
Newtonian fluids	When there is a linear relationship between fluid’s viscosity and shear stress. in Newtonian fluids, at a constant temperature, viscosity remains constant if shear stress increases.
Permeability (or absolute permeability)	The capacity of a porous media to transmit a fluid.
Photoresist	A light-sensitive material
Porosity	A ratio of void space to the total volume of the porous material e.g., rock.
Relative permeability	A dimensionless measure of the effective permeability of one fluid in presence of another fluid. It is the ratio of the effective permeability of that fluid to the absolute permeability.
Representative elementary volume	Is the smallest volume of a medium which its measured properties e.g., are representative of the whole medium.
Reynolds number	A dimensionless number which is the ratio of inertial forces to viscous forces within a fluid.
Segmentation	The process of partitioning a digital image into multiple segments in order to simplify the image and locate objects and boundaries.
Turbulent flow	When there are irregular changes in pressure and velocity of flow and in contrast to a laminar flow, there are vortices and eddies in the flow. A transition regime separates the laminar and the turbulent flows. This regime covers a wide range of Reynolds number. For fluid flow in pipes, turbulent flow happens when Re > 4000.
Voronoi diagrams (in computational geometry)	The partitioning of a 2D plane with a given set of vertices into convex polygons such that each polygon contains exactly one vertex from the set and every point in each polygon is closer to its vertex than to any other vertices.
Young-Laplace equation	Defines the capillary pressure across the interface between two static fluids.

## Symbols

ϕ	porosity
k	permeability
kr	relative permeability
Pc	capillary pressure
φ	sphere diameter
λ	wavelength
Ra	surface roughness
I	intensity
x	thickness
ϵ	linear attenuation coefficient
A	cross-sectional area
q	flow rate
µ	viscosity
ρ	density
∇P	pressure gradient
g	gravitational acceleration

## Abbreviations

2D	two dimensional
2.5D	two dimensional with variation in the third dimension
3D	three-dimensional
AM	additive manufacturing
CAD	computer aided design
CCD	charge coupled device
CLSM	confocal laser scanning microscopy
CMOS	complementary metal-oxide semiconductor
CTE	coefficient of thermal expansion
FDM	fused deposition modelling
FOV	field of view
HNA	solutions: hydrofluoric, nitric, acetic
ID	inside diameter
IR	image resolution
LOM	laminated object manufacturing
OD	outside diameter
PDMS	poly-di-methyl-siloxane; a type of polymer
PIV	particle image velocimetry
PMMA	poly-methyl-methacrylate; a type of polymer
PVA	polyvinyl alcohol
PVI	photoluminescent volumetric imaging
REV	representative elementary volume
RIM	refractive-index matching
SFL	stop-flow-lithography
SLA	stereolithography apparatus or simply stereolithography
SLM	selective laser melting
SLS	selective laser sintering
STL	standard tessellation language
